# Peptide Fingerprinting of Single Protein Molecules Using In‐Plane Nanopore Sensors Integrated to an Extended Immobilized Nanoscale Enzymatic Reactor (eINER)

**DOI:** 10.1002/smtd.71044

**Published:** 2026-09-16

**Authors:** Suresh Shivanka, Randy F. Espinal Cabrera, Maximillian Chibuike, Pramodi Vithanage, Indu A. Chandrasoma, Pubudu Premarathne, Matthew Verber, Steven A. Soper

**Affiliations:** ^1^ Department of Chemistry The University of Kansas Lawrence KS USA; ^2^ Center of BioModular Multiscale Systems for Precision Medicine USA; ^3^ University of North Carolina Chapel Hill NC USA; ^4^ Department of Mechanical Engineering The University of Kansas Lawrence KS USA; ^5^ Bioengineering Program The University of Kansas Lawrence KS USA; ^6^ KU Cancer Center University of Kansas Medical Center Kansas City KS USA

**Keywords:** nanopore sensing, peptide fingerprinting, plastic chips, single‐molecule detection

## Abstract

We report a thermoplastic nanofluidic device that combines an extended immobilized nanoscale enzymatic reactor (eINER) with a dual in‐plane nanopore time‐of‐flight (DNP‐ToF) reader for label‐free peptide fingerprinting of single protein molecules. As opposed to traditional membrane‐based nanopores, the DNP‐ToF reader uses two sub‐10 nm pores placed in series and are made from plastic to allow securing the resistive pulse sensing (RPS) independent ToF variable as well as the traditional RPS variables. The device enables on‐chip proteolytic digestion with downstream identification of peptide fragments. Using amyloid‐β (Aβ) as a model system, we demonstrate efficient digestion under nanoscale confinement consistent with a reaction‐limited regime and convection‐dominated transport. The device achieved robust event detection with >95% successful pairing of dual nanopore RPS signals enabling extraction of five key features (ΔI_1_/I_0_, ΔI_2_/I_0_, t_D1_, t_D2_, and ToF) from individual peptide events. Machine learning provided high‐confidence peptide identification with a random forest (RF) classifier achieving 100% accuracy and a cross‐validated accuracy of 99.6% ± 0.9%. Digestion efficiency was tunable through occupancy‐controlled conditions with RF‐assigned intact Aβ events decreasing from ∼9.5% at 1 nM to ∼0.33% at 100 pM. These results establish a scalable, high‐sensitivity platform for integrated single molecule analysis to secure proteomic data from mass‐limited samples.

## Graphical abstract

The protein Time‐of‐Flight (P‐ToF) chip can peptide fingerprint a single protein molecule by subjecting it to a solid phase endopeptidase cleavage reaction in‐line with a combination of resistive pulse sensing of the resulting single peptides and machine‐learning aided identification.

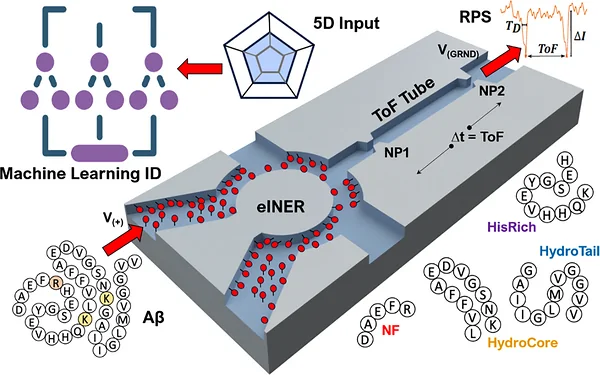

## 1 Introduction

Peptide fingerprinting (PF) is a fundamental approach for protein identification in which intact proteins are enzymatically cleaved into peptide fragments and the resulting peptide patterns compared with reference databases to identify the parent protein [1, 2]. Beyond protein identification, PF also plays a role in detecting post‐translational modifications (PTMs) [3], characterizing protein structures and conformational changes associated with disease progression, and studying protein–protein interactions [4].

The current gold standard for PF relies on liquid chromatography coupled with tandem mass spectrometry (LC‐MS/MS) [5]. Although LC‐MS/MS provides high‐resolution peptide identification, MS/MS measurements typically identify only a subset of the peptides present in a sample and can generate false‐positive peptide matches [6, 7]. Analysis of mass‐limited samples also remains challenging [8]. Modern high‐resolution LC‐MS/MS typically achieves a limit‐of‐detection (LOD) in the low femtomole range [9]. This becomes critical in a variety of clinical applications using liquid biopsy samples; it is not uncommon to secure a single circulating tumor cell (CTC) per mL of blood [10], which translates into ∼2 fmol of total protein, and with ∼20,000 proteins in the human proteome, generates ∼0.1 amol of each protein. This results in constraining proteome coverage limiting definitive identification of low abundance proteins.

Fluorescence‐based techniques have emerged as alternatives for studying proteins. Methods such as Förster resonance energy transfer (FRET), fluorescence correlation spectroscopy, and super‐resolution microscopy can provide detection even at the single‐molecule level [11]. However, these approaches require site‐specific labeling, are susceptible to photobleaching, and exhibit limited multiplexing capacity. In addition, fluorescence intensity does not directly encode intrinsic physicochemical properties and limits the ability for discrimination of peptide fragments in complex samples [12]. Collectively, these limitations indicate the need for alternative PF approaches that can improve the mass sensitivity to analyze low abundance proteins, but still enhance peptide coverage and simplify workflows with the potential for high throughput processing [13].

Nanopore technology enables label‐free, single‐molecule detection using resistive pulse sensing (RPS). In RPS, molecules translocating through a nanometer‐scale pore transiently disrupt a voltage‐biased ionic current generating current blockade events whose amplitude (ΔI) and duration (dwell time, t_D_) provide information about molecular properties such as size, charge, conformation, and so forth [14, 15, 16]. Nanopores are broadly classified as biological or solid‐state based on their origin [17]. Biological nanopores are naturally occurring protein pores, such as α‐hemolysin, and are embedded within thin membranes [18].

Solid‐state nanopores offer several advantages compared to biological pores including easily controllable pore geometry, mechanical robustness, chemical stability, and resistance to harsh environments [19, 20]. Based on their pore orientation relative to the substrate, solid‐state nanopores can be further classified into two categories: out‐of‐plane nanopores, in which the pore orifice is parallel to the substrate surface, and in‐plane nanopores in which the pore orifice is perpendicular to the substrate surface [21, 22]. In‐plane nanopores offer several advantages particularly their ability to be integrated into micro‐ and nanofluidic systems [23, 24]. In addition, multiple in‐plane nanopores can be arranged in series, increasing the amount of information obtained from individual molecules. For example, dual in‐plane nanopore (DNP) configurations enable measurement of the time‐of‐flight (ToF) of a molecule between two nanopores in addition to the conventional RPS parameters [21, 25]. ToF provides an additional molecule‐dependent parameter related to the apparent electrophoretic mobility (µ_app_) and is determined from a pair of correlated RPS events as the time difference between their occurrence [20]. Our group recently demonstrated that such a DNP‐ToF architecture can differentiate individual peptides using a combination of RPS and ToF [26].

However, relatively few studies have investigated the application of nanopores for PF, where proteins are digested and the resulting peptide fragments are detected and identified via RPS. In the studies reported to date, protein digestion was performed in bulk solution followed by introduction of the resulting peptide fragments into a biological nanopore for subsequent identification and PF [27, 28, 29]. Because protein digestion was performed in bulk solution, control over the digestion process was limited. In addition, bulk digestion can introduce issues such as enzyme autodigestion and potential sample loss, which may reduce data reproducibility [30, 31, 32].

We report herein a novel mixed‐scale fluidic system that uses solid‐state in‐plane nanopores integrated to a single‐protein proteolytic digestion for PF. The platform couples an extended immobilized nanoscale enzymatic reactor (eINER) with a solid‐state dual in‐plane nanopore reader (DNP‐ToF reader), which together comprise a *Proteomic Time‐of‐Flight* (P‐ToF) chip. Protein molecules undergo solid‐phase proteolytic digestion within the eINER and the resulting peptide fragments are subsequently transported to the DNP‐ToF reader for label‐free detection and identification. The reader provides five signal variables from each individual peptide, arising from distinct physicochemical characteristics, ΔI and t_D_ at each of the two nanopores together with the ToF. Collectively, these five signal variables provide a multidimensional peptide fingerprint, enabling high‐accuracy peptide identification through label‐free, single‐molecule detection. Importantly, protein digestion and subsequent peptide detection occur in near real time. Using this platform, the proteolytic digestion of a single protein molecule and the subsequent detection and identification of its proteolytically generated peptide fragments occur seamlessly eliminating the need for extensive sample handling.

As an initial proof‐of‐concept for P‐ToF operation, we used as the target protein Amyloid beta (Aβ) [33], which is strongly implicated in the pathogenesis of Alzheimer's disease [34]. Sensitive and selective detection of Aβ and investigation into its biophysiological properties in mass‐limited samples remains analytically challenging due to its low abundance and structural heterogeneity [35]. As a well‐characterized 40‐residue protein that yields a defined set of tryptic fragments [36], Aβ provides an ideal model for evaluating PF performance using P‐ToF. Using this model, we demonstrate integrated single‐molecule proteolysis and machine‐learning‐assisted identification of the resulting peptide fragments on the millisecond timescale for a single molecule input. This integrated architecture provides a framework for rapid PF of mass‐limited protein samples.

## 2 Results and Discussion

### 2.1 Fluidic Architecture and Multiscale Transport

The P‐ToF chip was fabricated from thermoplastics and consisted of two bonded layers, a substrate and cover plate (see Figure S1 for specific dimensions of functional units within P‐ToF). The substrate was COP 1060R and contained the complete mixed‐scale (nm → µm) fluidic network. The COC 8007 cover plate was thermally fusion bonded to enclose the fluidic channels; this combination of plastics produces a high process yield rate [20]. The fluidic structures in COP were produced from a silicon master via a resin intermediate using nano‐injection molding with an inter‐device relative standard deviation of <5% [37]. The final device measured 2 × 2 cm (Figure 1a) and contained 10 reservoirs with two reservoirs interconnected by a U‐shaped microchannel (5 µm × 5 µm), each feeding a microscale funnel region (10 µm → 1 µm width) patterned with pillars (Figure 1b). These funnel structures provided a gradual transition from micro‐ to nanoscale channels (sub‐100 nm), which provides high electrokinetic loading efficiency while minimizing abrupt field discontinuities that could otherwise promote molecular trapping [21]. The nanostructured components were made in Si via focused ion‐beam milling with a footprint of 20 × 20 µm (see Figure 1) while the microstructures were made via conventional photolithography and etching of the Si master. The resulting Si master could then be used to produce >100 resin stamps that were used for the injection molding; thus, each plastic device did not require photolithography and focused ion beam milling processing steps and could be injection molded in a single step at a production rate of ∼1000 devices per day [37].

**FIGURE 1:**
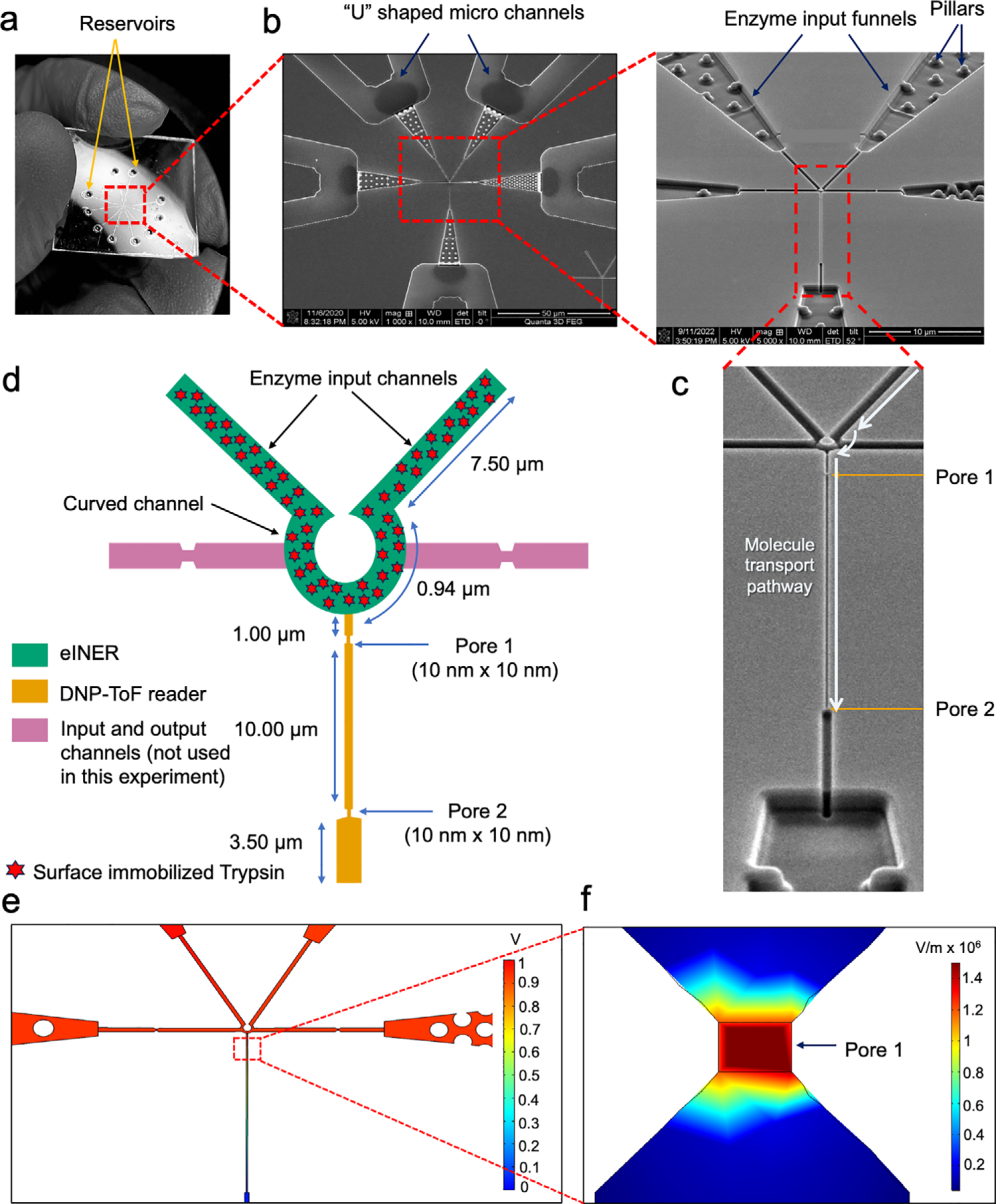
Architecture of the P‐ToF chip with its associated fluidic network. (a) Photograph of the complete thermoplastic P‐ToF chip (approximately 2 cm × 2 cm). The device contains ten reservoirs interconnected by five U‐shaped microchannels, each connecting a pair of reservoirs. (b) SEM of the silicon master highlighting the central nanofluidic region. This region contained funnel structures patterned with pillar arrays. Each funnel interfaces a U‐shaped microchannel on one side and a nanochannel on the other, enabling controlled transition from micro‐ to nanoscale dimensions. A total of five nanochannels converge toward the centrally located immobilized nanoscale enzymatic reactor (INER), which is formed as a curved chamber. (c) SEM of the sensing nanochannel incorporating the dual in‐plane nanopore time‐of‐flight (DNP‐ToF) reader. The nanochannel segment between the two nanopores functions as the flight tube (ToF channel), where time‐of‐flight measurements are performed. (d) Schematic representation of the eINER + DNP‐ToF reader (not to scale). The network was comprised of five nanochannels: two enzyme‐input nanochannels, one sample‐input nanochannel, one output nanochannel (these channels were not used in this set of experiments), and one sensing nanochannel. The extended INER (eINER) consisted of the INER (curved channel/chamber) and the two enzyme‐input nanochannels. The nanopore dimensions are 10 nm × 10 nm × 10 nm (length × width × depth); only the width and length are labeled in the figure. (e) COMSOL‐simulated electric potential distribution across the nanofluidic network under an applied voltage. (f) Corresponding electric field magnitude (V m^−^^1^) showing strong field localization within the constricted sensing region (nanopore), illustrating geometric field amplification.

P‐ToF carried out single‐peptide detection and identification using the DNP‐ToF reader, which consisted of two in‐plane nanopores separated by a nanochannel flight tube (Figure 1c). Molecules were detected by RPS at each nanopore, and the delay time between the appearance of two consecutive RPS signals was measured to obtain ToF information. Proteolytic digestion occurred within a nanoscale chamber (eINER) at the center of P‐ToF with trypsin covalently immobilized not only within this circular‐shaped chamber but also along the two enzyme‐input channels forming a continuous enzyme‐functionalized pathway within the eINER (Figure 1d). This configuration created a confined reaction corridor in which a substrate interacted repeatedly with surface‐bound enzymes during electrokinetic transport.

### 2.2 Nanoscale Transport and Electric Field Hierarchy

P‐ToF was engineered to possess a hierarchical micro‐to‐nanofluidic architecture that efficiently guided biomolecules from macroscopic reservoirs into nanoscale reaction and sensing regions and confined high electric fields within the DNP‐ToF reader [38]. More importantly, the P‐ToF chip permitted online detection of reaction products from a single molecule generated within the eINER and did so in near real time (see SI, Section 1).

To understand the molecular transport and electric‐field distribution in P‐ToF, voltage drops and electric fields were calculated along the voltage biased pathway (Figure S1 and Table S1), which included both in‐plane nanopores. This path consisted of a 7.5 µm enzyme‐input channel and a 0.94 µm hemispherical INER segment each with 250 × 250 nm^2^ cross‐sections, followed by the sensing pathway composed of a 1 µm pre‐first‐pore segment (50 × 50 nm^2^), a 10 nm‐long in‐plane nanopore (hemispherical cross section with a 10 nm apparent diameter), a 10 µm flight tube (50 × 50 nm^2^), a second 10 nm‐long in‐plane nanopore (same cross section as the first nanopore in the series), and a 3.5 µm post‐second‐pore segment expanding to 250 × 250 nm^2^ (Figure S1).

The 250 × 250 nm^2^ regions experienced low electric fields (∼33 Vcm^−1^) compared to the DNP‐ToF reader. Upon transition into the 50 × 50 nm^2^ DNP‐ToF reader, the average electric field was ∼8.3 × 10^2^ Vcm^−1^, approximately 25× higher than that in the curved segment of the eINER, which served to increase the efficiency of transfer of digestion products from the eINER into the DNP‐ToF reader [39]; a discussion on the rationale for selecting these dimensions can be found in Section 4.1 of the SI.

Although each in‐plane nanopore is only 10 nm long, its small cross‐section forced a measurable fraction of the applied voltage to drop across the pore, yielding average electric fields of ∼2.1 × 10^4^ Vcm^−1^. This strong local field drives high molecular capture and rapid translocation minimizing residence time at the pore entrance [40]. These conclusions were supported by COMSOL simulations: Figure 1e illustrates voltage division along the nanoscale transport pathway, while Figure 1f highlights the high electric‐field localization within the nanopore region. In addition to high electric‐field localization, nanoscale pores can exhibit concentration polarization and/or ion current rectification due to surface charge and geometric confinement. These effects occur primarily at the nanopore and not in the larger nanochannel enhancing local electric‐field gradients near the pore entrance, thereby improving molecular capture and transport through the pore [41, 42, 43]. In addition, we adjusted the UV/O_3_ activation time (i.e., 6 min; see Section 6 in the SI) to keep the surface charge low to reduce the effects of concentration polarization at the in‐plane nanopore entrances. Small in‐plane nanopores also increase the signal‐to‐noise ratio for RPS detection of the peptides [26].

### 2.3 EINER Coupled to the DNP‐ToF Reader

Trypsin (737 nM solution) was immobilized within the eINER using EDC/NHS coupling chemistry [44]. Site‐selective enzyme immobilization was achieved electrokinetically by applying a bias voltage between the two enzyme‐input channels (Figure 2a). UV/O_3_ treatment of the thermoplastic generated oxygen‐containing functional groups including hydroxyl, aldehyde, ketone, and carboxyl (–COOH) groups with the latter serving as reactive sites for covalent enzyme attachment to the plastic surface (Figure 2b). The attachment occurs between the ‐COOH groups on the surface of activated plastic and endogenous primary amines found in the enzyme to produce a stable amide bond.

**FIGURE 2:**
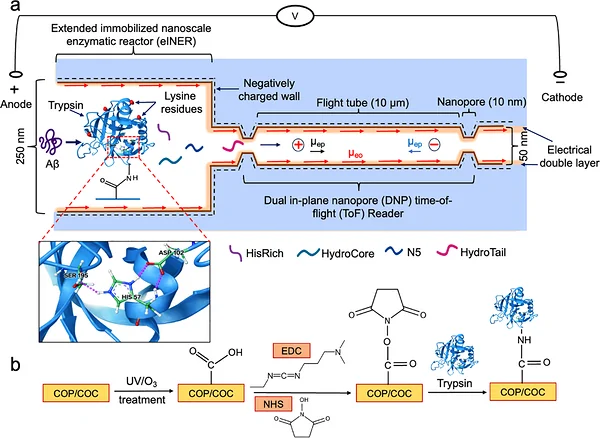
Integration of the eINER with the DNP‐ToF reader comprising P‐ToF. (a) Protein molecules (here, Aβ) are introduced into the nanoscale bioreactor and undergo proteolytic digestion as they traverse the reactor where trypsin is covalently immobilized using UV/O_3_‐generated surface carboxyl groups of the thermoplastic. The proteolytically generated peptide fragments are subsequently directed toward the DNP‐ToF reader for detection and identification. Within the flight tube, molecular transport is governed by the electrophoretic mobility (µ_ep_) and electroosmotic flow (µ_eo_). Electroosmotic flow is directed from the anode to the cathode and carries all peptides to the cathode irrespective of charge. The enlarged inset of trypsin highlights the catalytic triad (Ser195, His57, and Asp102), which constitutes the active site. The flight tube length is 10 µm with a cross section (rectangular) of 50 × 50 nm^2^. Each nanopore is 10 nm in length and hemispherical in shape with a 10 nm apparent diameter. The eINER has a cross‐section of 250 nm × 250 nm. The schematic is not drawn to scale. (b) Schematic illustration of trypsin immobilization onto the UV/O_3_ activated thermoplastic surface via EDC/NHS coupling chemistry. For specific dimensions, transport properties, and sampling volume of the eINER, see SI, Sections 2 and 3.

To assess whether enzyme activity was preserved following surface immobilization, the catalytic mechanism and structural accessibility of immobilized trypsin within the eINER was evaluated (Figure S2). Trypsin possesses a canonical catalytic triad (Ser195, His57, and Asp102) within a solvent‐accessible cleft enabling peptide‐bond hydrolysis via a charge‐relay mechanism. Covalent immobilization occurs predominantly through surface‐exposed lysine residues, which are spatially distributed away from the catalytic site, minimizing steric interference of substrate access following immobilization to the thermoplastic surface. This structural arrangement preserves both catalytic function and substrate specificity supporting efficient proteolytic activity within the eINER.

We carefully characterized the immobilization of trypsin within the eINER using EDC/NHS chemistry, which couples the plastic surface UV/O_3_ generated ‐COOH groups with the lysine residues intrinsically found in trypsin (∼14 lysine residues distributed throughout the solvent‐exposed surface loops of trypsin; see Figure S3a) [45]. Surface characterization after immobilization was interrogated using AFM, which revealed an average enzyme height of 4.76 ± 0.36 nm, consistent with literature values for trypsin in its catalytically competent conformation (Figure S3b–d). Two‐dimensional COMSOL simulations confirmed that the resulting electric‐field distribution was confined within the eINER minimizing leakage into adjacent input, output, and sensing nanochannels (Figure S3e). For information on the trypsin surface load within the eINER, please see SI, Section 16.

Following immobilization, Aβ was introduced through the enzyme‐input channels and electrokinetically transported through the eINER. During transit, digestion occurs in a distributive and non‐ordered manner. Aβ contains one arginine (Arg5) and two lysine residues (Lys16 and Lys28), resulting in three possible cleavage sites and four peptide fragments: N5, HisRich, HydroCore, and HydroTail (Table S2) [46]. As a distributive enzyme, trypsin performs a single cleavage per binding event. Accordingly, an Aβ molecule binds to an immobilized trypsin, undergoes one cleavage, dissociates, and then continues downstream where it may encounter an additional immobilized enzyme for subsequent cleavage [46].

After digestion, the resulting peptide fragments are shuttled into the DNP‐ToF reader of P‐ToF. This single‐molecule electrokinetic transport is efficiently driven by the asymmetric electric‐field distribution between the eINER and the DNP‐ToF reader unit of P‐ToF. The higher electric field in the DNP‐ToF reader efficiently pulls molecules from the eINER into this element. Due to differences in the electric field strength between the eINER and the DNP‐ToF reader, trypsin‐generated peptides tend to slightly drift within this reactor prior to entering the DNP‐ToF reader preventing simultaneous entry of all peptides to allow single‐peptide processing in a predominately sequential process (see Figure S1).

Nanoscale confinement within the eINER enhances digestion efficiency by minimizing diffusional distances between the flowing substrate and the enzyme‐coated walls [47]. The rectangular nanochannel geometry provides four enzyme‐functionalized surfaces and cleavage occurs when Aβ diffuses from the bulk flow toward the catalytic interfaces. Immobilization eliminates trypsin autodigestion relative to solution‐phase reactions and maintains a stable surface density of active enzyme improving reproducibility of the digestion process [48].

Molecular transport within the device is governed by electrophoresis, electroosmosis, and molecular diffusion. Electrophoresis describes the migration of charged peptides under an applied electric field, while the electroosmotic flow (EOF) corresponds to bulk liquid motion within the charged nanochannel—both of these are longitudinal transport processes. The apparent molecular electrophoretic mobility (µ_app_) is the vector sum of the intrinsic electrophoretic mobility (µ_ep_) and electroosmotic mobility (µ_eo_) (Figure 2a) [26, 49]. Under the experimental conditions (pH 7.4, 1 V), HydroTail is neutral, whereas Aβ and the other fragments carry negative charges. Experimentally, all species were observed to migrate in the direction of the EOF indicating that µ_eo_ dominates over µ_ep_ for negatively charged peptides and Aβ under these conditions (see below for a discussion on how µ_app_ values for Aβ and the peptides were calculated from the ToF data).

The relative contributions of convection and diffusion can be described by the Péclet number (Pe). Using the transport parameters of the eINER, Pe ≈ 127 indicating that transport is dominated by convection under the applied voltage (see SI, Section 7). Thus, Aβ molecules are carried downstream primarily by the EOF. Because the nanochannel cross section is 250 × 250 nm^2^, diffusion from the bulk flow to the enzyme‐functionalized wall allows for a high encounter rate between the substrate molecules to reach the immobilized enzyme surface.

### 2.4 Reaction‐Limited Digestion Under Occupancy‐Controlled Nanofluidic Transport

Enzymatic digestion within the eINER was analyzed using a reaction‐transport‐occupancy framework. Under convection‐dominated transport described above, analyte molecules are carried downstream electrokinetically. The nanoscale dimensions of the eINER channels (250 nm × 250 nm) reduce diffusional distances between the flowing analyte and the enzyme‐functionalized wall. Damköhler analysis (Da ≪ 1; see SI, Section 8) indicated that intrinsic trypsin turnover is reaction‐limited rather than diffusion‐limited under the applied electrokinetic transport conditions [50]. Under these conditions, nanoscale confinement increases the likelihood of analyte encounters with the enzyme‐functionalized wall enabling substrate molecules to efficiently access immobilized enzyme during transport through the reactor.

Because the Debye length under the high‐ionic‐strength buffer conditions used here (1× PBS with 1 M KCl) is much smaller (λ_D_ ≈ 0.29 nm) than the 250 nm channel dimension (see SI, Section 9), overlap of the electric double layers (EDL) is negligible and EOF transport can be approximated as plug‐like. Under this approximation, analyte enzymatic conversion (*[A]_o_*) follows:

(1) $${\left[A\right]}_{o}={\left[A\right]}_{i}\;\exp\left(-14.43\;\frac{D_{A}F_{A}\;l}{v\;d^{2}}\right)$$

where analyte molecules enter the reactor at [A]*_i,_* D_A_ = 1.0 × 10^−10 ^m^2^s^−1^ is the analyte diffusion coefficient, F_A_ is the active fraction of the surface immobilized enzymes, l = 8.44 µm is the reactor length, d = 250 nm is the channel diameter, and v = 1.5 × 10^−3 ^m s^−1^ is the linear velocity. For these experimentally relevant parameters, the model predicts high single‐pass conversion probabilities. Importantly, this expression reflects geometry‐dependent enzyme‐substrate encounter statistics rather than assuming diffusion‐limited catalysis. The SI further evaluates the digestion probability using the dimensionless reaction‐opportunity parameter µ = 14.43(D_A_​F_A_​l)/(vd^2^), which captures the cumulative number of enzyme‐substrate encounters during electrokinetic transit. As shown in Figure S4, the probability of achieving complete digestion (≥3 cleavage events) approaches unity as µ increases, and the experimentally relevant value falls within a regime where near‐complete digestion is predicted (Table S3). Because µ ∝ (vd^2^)^−1^ (see SI, Section 10), both longitudinal velocity and channel diameter strongly influence digestion efficiency (see SI, Sections 11 and 12). This dependence is illustrated in Figure S5, where smaller channel diameters (50–250 nm) maintain a high probability of complete digestion across a broad velocity range, whereas larger channels (>500 nm) show a rapid loss of digestion probability. The corresponding conversion efficiency predicted by the plug‐flow reaction model is shown in Figure S6, which demonstrates that nanoscale channels (50–250 nm) yield high enzymatic conversion even at the experimentally relevant velocity, while micrometer‐scale (0.5–2 µm) channels exhibit a pronounced decrease in conversion due to reduced enzyme‐substrate encounter frequencies.

### 2.5 Multivariate Fingerprinting of Peptides Based on Physicochemical Signatures

For PF generation, Aβ and its four trypsin‐generated fragments (N5, HisRich, HydroCore, and HydroTail) were individually electrokinetically transported through the P‐ToF chip in the absence of trypsin within the eINER; this allowed for collection of training data for the machine learning models evaluated below. Electrodes were connected to one reservoir (labeled as +1 V, anode) linked to the enzyme input channels and to another reservoir (labeled as GRND, cathode) connected to the terminal end of the DNP‐ToF reader. Aβ molecules entered through an enzyme‐input channel, traversed the eINER, and were subsequently directed into the DNP‐ToF reader (Figure 3a). As each molecule passed through the DNP‐ToF reader, it generated two current blockade events referred to as a peak pair (Figure 3b). Peak pairs were assigned using predefined selection criteria implemented in MATLAB (see SI, Section 5). RPS events that did not satisfy these criteria were classified as unpaired and not included in the data analysis. Because the same peak‐detection and pairing criteria were applied consistently to all peptide classes, exclusion of the small fraction of unpaired events is not expected to introduce a systematic class‐dependent selection bias. These events are indicated in the RPS traces as blue stars (Figure 3b). For all peptides, >95% of detected events were successfully paired, confirming the robustness of the peak‐pairing algorithm and indicating low probability of simultaneous multi‐molecule occupancy within the DNP‐ToF reader.

**FIGURE 3:**
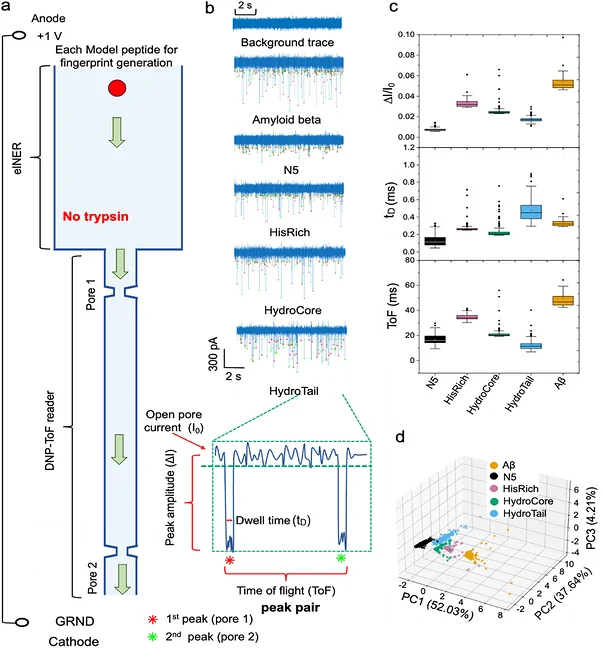
Fingerprint generation by electrokinetic transport of model peptides through the P‐ToF chip. (a) Each of the five model peptides, Aβ, N5, HisRich, HydroCore, and HydroTail were electrokinetically transported through the eINER and the DNP‐ToF reader in the absence of trypsin (one device per peptide). All peptides migrated from the anode (+) to the cathode (ground). (b) Representative current traces for a blank solution (1× PBS, 1 M KCl) and for each peptide. As each peptide translocated through the DNP‐ToF reader, it generated paired current blockade events (peak pairs). Five primary variables were extracted from each peak pair: dwell time (t_D_ for pores 1 and 2), peak amplitude (ΔI for pores 1 and 2), and ToF. The representative peak pair shown corresponds to HydroTail. Red and green stars denote the first and second events of a peak pair, respectively, while blue stars indicate unpaired events in the current trace. The baseline current corresponds to the open‐pore current (I_0_). All data were collected at 1 nM peptide concentration, 1 V applied voltage, pH 7.4 (1× PBS, 1 M KCl), a sampling rate of 250 kHz, and a 10 kHz low‐pass filter. (c) Box plots showing the distributions of normalized peak amplitude (ΔI/I_0_), dwell time (t_D_), and ToF for the five peptides. The center line represents the median, the boxes indicate the 25th–75th percentiles, and the whiskers extend to 1.5× the interquartile range. Individual data points denote outliers. (d) Three‐dimensional (3D) principal component analysis (PCA) score plot generated using ΔI/I_0_, t_D_, and ToF as input features. For this data, the ΔI/I_0_, t_D_ values for pores 1 and 2 were plotted together. PC1, PC2, and PC3 account for 52.03%, 37.64%, and 4.21% of the explained variance, respectively, corresponding to a cumulative explained variance of 93.88%.

Although molecules traverse the two nanopores in series, both translocation events may not necessarily produce detectable peaks. As previously reported for dual‐nanopore measurements, the primary origins of unpaired peaks are a missing detectable translocation event at one of the two nanopores and/or the presence of more than one molecule within the flight column. Previous work using stacked nanopores similarly reported that 94% of detected peaks could be unambiguously assigned to translocating dsDNA [51]. Missing peaks may result from molecular dimensions relative to the nanopore geometry and the shallow V‐shaped geometry [52] of the in‐plane nanopores [53], which can cause variations in transient current signals depending on the translocation location within the nanopore. In addition, sufficiently fast translocation events may not be detected within the 10 kHz bandwidth of the current amplifier [54, 55]. Overlapping molecules within the flight column can also prevent unambiguous peak‐pair assignment.

From each event pair, five variables were extracted: normalized peak amplitude (ΔI/I_0_) for pores 1 and 2, dwell time (t_D_) for pores 1 and 2, and the ToF. Among these, the ΔI/I_0_ and t_D_ are RPS‐dependent variables while the ToF is related to the µ_app_ of the peptide and thus, RPS independent. Because the in‐plane nanopores for the DNP‐ToF reader were similar in size, ΔI/I_0_ and t_D_ were grouped together for the following analysis. Box plots revealed distinct distributions for each peptide across all parameters (Figure 3c). These distinctions arise from differences in the physicochemical properties of the peptides generated from trypsin action on Aβ (Table S2).

ΔI/I_0_ is primarily governed by hydrodynamic size of the biomolecule. Aβ exhibits the largest ΔI/I_0_ due to its larger hydrodynamic radius (R_h_), which displaces a greater electrolyte volume during translocation and thus produces a larger transient current drop [56]. N5, which is more than threefold smaller than Aβ, correspondingly exhibited a smaller ΔI/I_0_ value. The remaining fragments followed this same size‐dependent trend. Notably, even sub‐nanometer differences in R_h_ among HisRich, HydroCore, and HydroTail were resolvable in terms of their respective ΔI/I_0_ values demonstrating the sensitivity of our in‐plane nanopores.

t_D_ reflects a combination of hydrophobic wall interactions and hydrodynamic drag. Hydrophobicity of the peptide fragments was evaluated using GRAVY scores (Table S2); more positive values indicate greater hydrophobicity [57]. Hydrophobic peptides can transiently interact with hydrophobic pore regions, increasing the residence time within the in‐plane nanopore. As expected, HydroTail, the most hydrophobic fragment showed the longest t_D_. However, t_D_ does not strictly follow GRAVY ranking indicating additional contributions. Hydrodynamic drag (F_drag_), which scales with R_h_ according to Stokes’ law, plays a role as well. Aβ exhibited a longer t_D_ than HydroCore despite its lower hydrophobicity because its larger size increases viscous resistance. Conversely, the smallest fragment, N5, showed the shortest dwell time due to minimal drag. HisRich displays slightly longer t_D_ than HydroCore, which may arise from transient electrostatic interactions between its positively charged histidine residues and the negatively charged nanopore surface.

ToF is governed primarily by µ_app_ in the flight tube, which represents the vector sum of µ_ep_ and µ_eo_. Under our experimental conditions (pH 7.4, 1 V), EOF dominates transport. Negatively charged peptides experience electrophoretic drag opposing the EOF increasing their ToF, whereas neutral HydroTail migrates essentially at the EOF and exhibited the shortest ToF. This behavior is clearly reflected in the data; HydroTail showed the smallest ToF, while highly negatively charged Aβ exhibited the longest. Although HisRich (−0.9) and HydroCore (−1.0) have roughly similar net charges, HisRich displays a longer ToF likely due to greater hydrodynamic drag and possible transient interactions of histidine residues with the negatively charged flight tube walls. Interestingly, despite having the second‐highest net negative charge, N5 shows a relatively small ToF, indicating that for small peptides hydrodynamic drag dominates over electrophoretic effects.

Using the ToF for HydroTail (11.4 ± 3.9 ms) and assuming negligible wall interactions, the EOF in the flight tube was estimated to be 1.0 ± 0.3 × 10^−^^4^ cm^2^ V^−^^1^ s^−^^1^; we note that this value neglects potential wall interactions that may influence µ_app_ of HydroTail and thus, this value of the EOF should be viewed as provisional. However, we could calculate the apparent electrophoretic mobilities of the tryptic peptide fragments of Aβ using µ_app_ = *l/(E × ToF)*, where *l* is the flight tube length (10 µm) and *E* is the electric field strength in the flight tube (835 V/cm). Table S2 in the SI contains the µ_app_ values for Aβ and its tryptic digestion products. Because the channel walls are negatively charged, the EOF moves from anode to cathode. As can be seen from the data in Table S2, the EOF was greater than the µ_app_ values for all peptide fragments except in the case of HydroTail, which was equal to the EOF. Therefore, the EOF was sufficient to carry all peptide fragments and Aβ toward the cathode.

### 2.6 Machine Learning for Single Peptide Identification

3D PCA analyses captured 93.9% of the cumulative variance demonstrating that the three primary features (ΔI/I_0_, t_D_, and ToF) contained sufficient discriminatory information to primarily differentiate the peptides (Figure 3d). However, PCA is an unsupervised dimensionality‐reduction method and thus, it does not provide definitive class assignment required for PF. Therefore, to achieve robust and quantitative peptide identification, we evaluated multiple supervised machine learning (ML) algorithms and selected the most appropriate model based on classification performance.

Five feature variables were extracted from each peak pair—in this case, the ΔI/I_0_ and t_D_ values for pore 1 and 2 were used as separate inputs into the machine learning model as well as the ToF. These features were introduced into multiple supervised classification machine learning models including linear discriminant analysis (LDA), quadratic discriminant analysis (QDA), support vector machines (SVM), multilayer perceptron (MLP), and random forest (RF) classifiers (Figure 4a).

**FIGURE 4:**
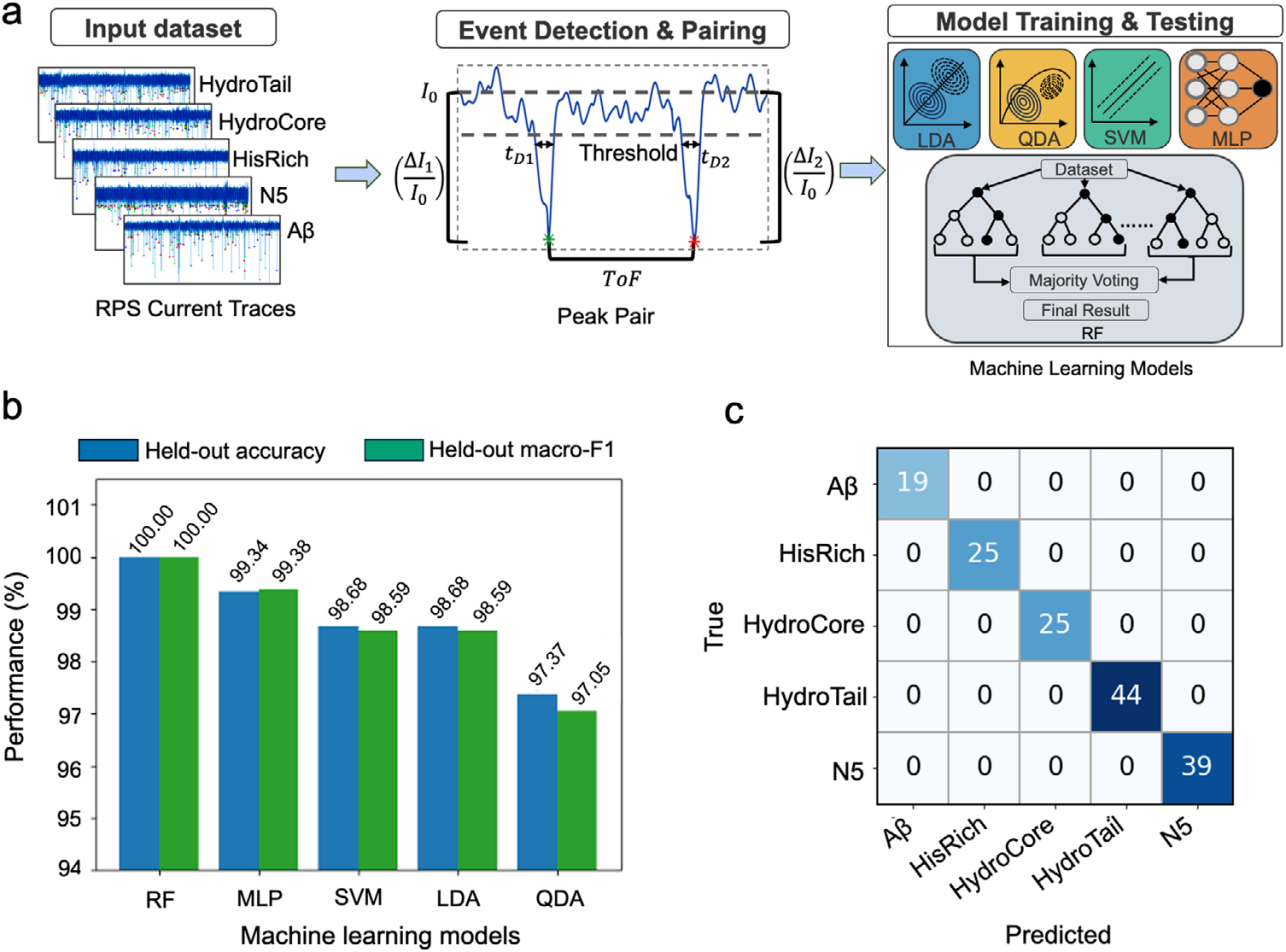
Performance of the P‐ToF chip for peptide fingerprinting using machine learning classification. (a) Workflow of the machine learning training and testing procedure. Five current traces with RPS events of each peptide including the intact protein (Aβ, N5, HisRich, HydroCore and HydroTail) were used as the input dataset and analyzed using the in‐house EventFinder v2.55 software to extract peak pairs and their associated features. A total of 758 peak pairs were obtained. Extracted features included ToF, dwell times for pore 1 (t_D1_) and pore 2 (t_D2_), calculated as full width at half maximum (FWHM), and normalized peak amplitudes for pore 1 (∆I_1_/I_0_), and pore 2 (∆I_2_/I_0_), These five features were used to train and evaluate the machine learning models. An 80/20 train‐test split was implemented and model robustness was further assessed using 5‐fold stratified cross‐validation. (b) Comparison of classification performance across the evaluated models. Bars represent held‐out accuracy and held‐out macro‐F1 values. (c) Confusion matrix of the random forest (RF) model evaluated on the held‐out test set (20% of total data, *n* = 152 events), showing no misclassification among the five training classes. All algorithms achieved high accuracy (>96%). RF yielded the highest held‐out accuracy (100%) and held‐out macro‐F1 score (100%), followed by multi‐layer perceptron (MLP), support vector machines (SVM), linear discriminant analysis (LDA), and quadratic discriminant analysis (QDA).

The unequal numbers of peak pairs among peptide classes resulted from the numbers of experimentally recorded events satisfying the same predefined peak‐pair selection criteria within a fixed acquisition period. No oversampling, undersampling, or class weighting was applied. Stratified data splitting was used to preserve class representation during model training and evaluation.

The classifier was trained and evaluated using peptide fingerprints acquired under the defined experimental conditions of this study, and cross‐device or cross‐condition generalization was not evaluated.

Model performance was evaluated using held‐out test accuracy and held‐out macro‐F1 scores to assess overall and class‐balanced classification performance [58]. Macro‐F1 gives equal weight to the class‐specific F1 scores regardless of class size, whereas weighted‐F1 weights the class‐specific scores according to class size [59, 60]. Weighted‐F1 and balanced accuracy were additionally calculated to evaluate the potential influence of the unequal class sizes (see SI, Section 13). Among the evaluated models, the RF classifier demonstrated the strongest overall performance achieving 100% for both held‐out accuracy and held‐out macro‐F1 scores (Figure 4b). The corresponding confusion matrix showed no systematic misclassifications confirming that the extracted P‐ToF features provided sufficient discriminatory power for reliable PF (Figure 4c). The superior performance of the RF model likely arises from its ensemble‐based structure, which effectively captures nonlinear feature interactions while maintaining robustness against experimental variability and moderate dataset size [61]. Consequently, all subsequent classification analyses were performed using the RF model.

It should be noted that peptides other than the Aβ‐derived fragments can also translocate through the DNP‐ToF reader and generate RPS signals (see Table S2 in the SI). Therefore, peptide specificity is achieved through the combined multidimensional signature of ΔI_1_/I_0_, ΔI_2_/I_0_, t_D1_, t_D2_, and ToF rather than through selective nanopore capture. In complex samples, peptides with similar physicochemical properties may produce overlapping signal distributions and represent potential interferents. Expansion of the machine‐learning training library to include additional peptide species and relevant interferents would therefore be important for future analysis of complex proteomic samples.

Feature importance analysis further supported the robustness of the RF model (Figure S7). Permutation importance indicated that ToF was the dominant feature governing model generalization, followed by normalized peak amplitudes, while dwell times contributed minimally. Additional validation using label‐shuffling yielded near‐random accuracy (23.7 ±1.9%), confirming that the high classification performance arises from learned feature structure rather than overfitting.

To decouple intrinsic enzymatic capability from stochastic multi‐molecule effects, digestion was performed at 1 nM and 100 pM Aβ concentrations. Based on the eINER volume (∼1.06 fL, see SI, Section 14), Poisson occupancy analysis yielded λ ≈ 0.64 at 1 nM and λ ≈ 0.064 at 100 pM (see SI, Section 15 for occupancy probability at different concentrations) [62]. These corresponded to multi‐occupancy probabilities P (≥2) of 13.4% and 0.19%, respectively (Table S4).

At 1 nM, 9.50% of accepted events were assigned by the RF classifier to the intact Aβ class, whereas at 100 pM this fraction decreased to 0.33% (Figure 5a,b, Table S5 and SI, Section 17). These values therefore represent RF‐assigned intact Aβ event fractions rather than an independent biochemical measurement of undigested Aβ. Because intrinsic enzyme kinetics and transport conditions were unchanged between experiments, this difference arises from occupancy‐driven effects rather than limitations in catalytic capacity. Under multi‐occupancy conditions, transient competition for enzyme active sites and residence‐time dispersion increase the probability that at least one molecule exits the eINER incompletely digested. In contrast, the 100 pM regime operates almost exclusively under single‐molecule occupancy allowing the reaction‐limited enzymatic process to approach its intrinsic efficiency.

**FIGURE 5:**
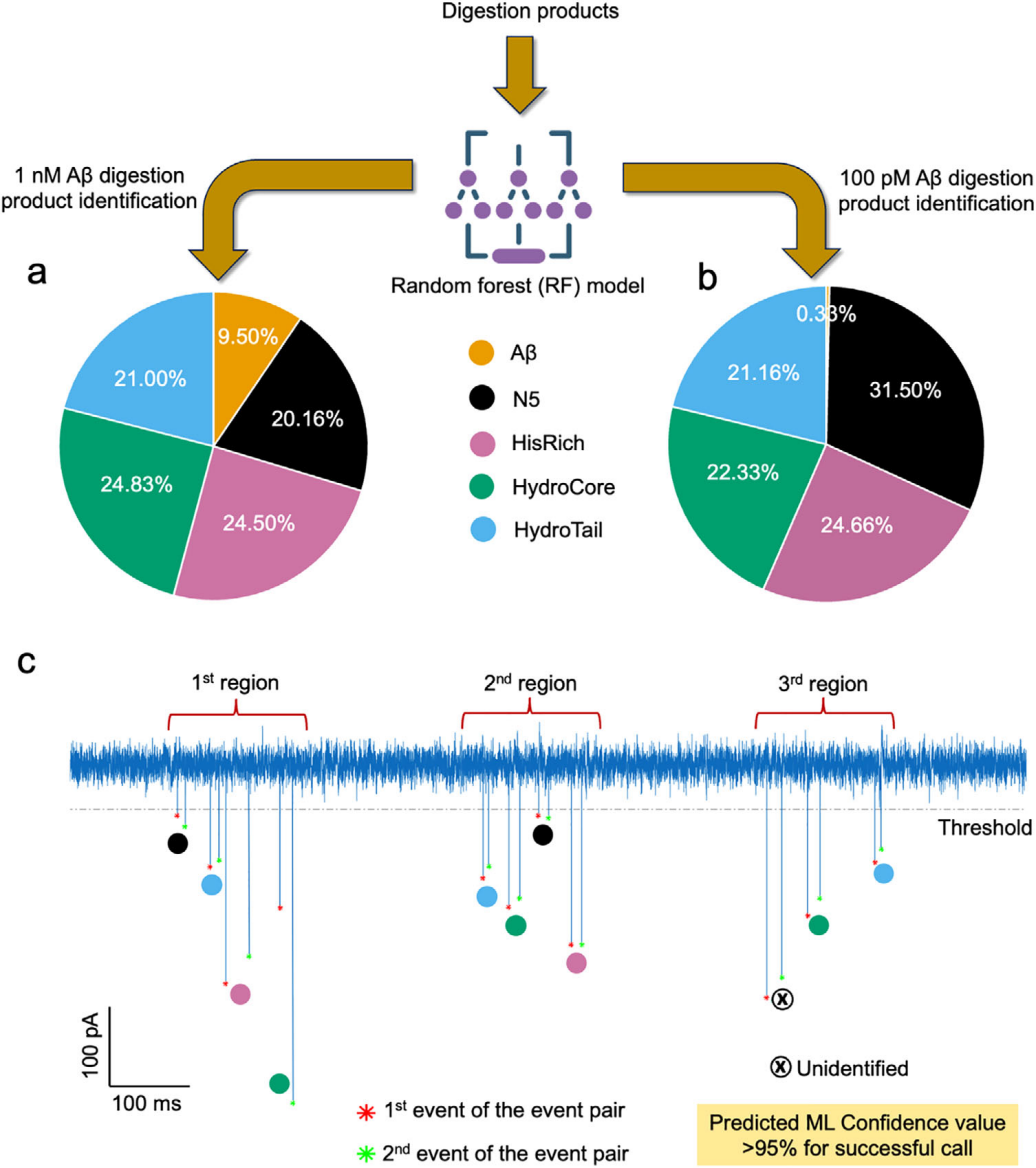
Application of the trained random forest (RF) model for the identification of digestion products. (a,b) Pie charts showing the relative percentages of different peptide fragments generated following the digestion of Aβ at concentrations of 1 nM and 100 pM. (c) Representative 1100 ms RPS current trace collected from the digestion of 100 pM Aβ. Three regions corresponding to three single‐molecule Aβ digestions were identified. The first and second regions contain four peak pairs, whereas the third region contains three peak pairs. Red and green stars denote the first and second events within each peak pair, respectively, assigned using the EventFinder v2.55 software based on predefined peak‐pair selection criteria. Each peak pair was evaluated using the RF model, with most assignments showing high classification confidence; the low‐confidence 58.8% event is discussed in the main text as an unassigned species that may be consistent with an untrained digestion intermediate.

Idealized complete digestion would yield equal fragment percentages of (100–%undigested)/4. At 1 nM this corresponded to 22.6% per fragment, and at 100 pM 24.9% both of which deviate modestly from theoretical predictions (Figure 5a,b). This deviation is not indicative of diffusion‐limited digestion, rather it reflects the formation of partially cleaved intermediates and their projection onto the discrete labels used in the RF classifier. Because the RF model was trained only on intact Aβ and the four fully cleaved peptide fragments, intermediate species are necessarily mapped onto the nearest class in feature space often with reduced confidence scores.

Direct single‐molecule analysis of a 1100 ms RPS current trace collected at 100 pM further confirmed this interpretation (Figure 5c). Three temporally separated digestion regions were identified; the first and second regions contained four peak pairs, whereas the third region contained three peak pairs with each region corresponding to single‐molecule Aβ trypsin‐mediated digestion products. In the first and second regions, the four expected fragments (N5, HisRich, HydroTail, and HydroCore) were detected in each region with no intact Aβ assignments. In the first digestion region, N5, HydroTail, HisRich, and HydroCore were identified with classification probability of 100%, 100%, 100%, and 98.4%, respectively. In the second digestion region, HydroTail, HydroCore, N5, and HisRich were identified with confidence levels of 100%, 99.8%, 100%, and 100%, respectively. However, in the third region, the first peak pair was identified as HydroCore but with a low confidence level of 58.8%, whereas the other two peak pairs were identified as HydroCore and HydroTail with confidence levels of 100% and 97.2%, respectively. The 58.8% assignment was therefore treated as a low‐confidence, unassigned species that may be consistent with an untrained digestion intermediate. Unsupervised clustering could be used in future studies to determine whether such low‐confidence events form reproducible groups in the measured feature space, although independent standards would be required to establish sequence or cleavage state and to expand the supervised training set.

## 3 Conclusion

We have developed an integrated mixed‐scale fluidic system that combines solid‐phase proteolysis with dual in‐plane nanopore time‐of‐flight detection and identification for single‐molecule PF. By coupling an eINER with a DNP‐ToF reader, the fluidic system enables continuous on‐chip digestion, electrokinetic transport, and label‐free detection and identification of peptides from a single protein input. With this configuration, mixtures of mass limited protein samples can be analyzed at the single‐molecule level and identified with high accuracy even for low abundance species.

Under nanoscale confinement, enzymatic digestion proceeded in a reaction‐limited regime in which encounter statistics and stochastic molecular occupancy governed conversion efficiency. The nanoscale geometry of the reactor promoted enzyme‐substrate interactions during electrokinetic transport, enabling efficient single‐pass digestion prior to nanopore sensing.

The DNP‐ToF reader provided multivariate readout by simultaneously measuring ΔI/I_0_, t_D_, and ToF, each reflecting distinct physicochemical properties of the peptides. These complementary signal variables generated multidimensional molecular fingerprints that enabled reliable identification of peptide fragments at the single‐molecule level. When combined with supervised machine learning, these features provided highly accurate peptide classification.

Analysis of individual digestion events indicated that complete enzymatic digestion of a single molecule occurred on a timescale of ∼150 ms, highlighting the rapid kinetics enabled by nanoscale confinement and electrokinetic transport. This fast processing, combined with the label‐free multivariate readout of the DNP‐ToF reader, establishes a framework for high‐speed single‐molecule PF even for mass limited samples.

Beyond this proof‐of‐concept demonstration, the architecture of the P‐ToF platform enables straightforward scaling by integrating multiple P‐ToF units in parallel for high throughput processing. For example, with the current footprint of the FIB‐milled region of P‐ToF, we can fit ∼1000 P‐ToF devices per 4″ wafer. Thus, because a single protein can be PF in ∼150 ms, we can potentially process 576,000 samples per day using a single device or 576 million per day in the multichannel format. As a comparison, standard benchtop LC‐MS/MS techniques can PF a protein sample in ∼16 h per sample.

In terms of proteomic analysis, while the present example required training on each peptide with a known sequence of the proteolytic digestion of Aβ, this could be intractable when dealing with the human proteome with >20,000 different proteins generated from the human genome (>400,000 different proteins if one includes splice variants and post‐translational modifications). This challenge may be addressed by determining the amino acid composition of each peptide fragment resulting from an unknown protein subjected to PF. For example, an ongoing extension in our laboratory is the integration of a second DNP‐ToF reader with an INER containing an exopeptidase, which could enable amino‐acid‐level analysis and peptide identification by comparison with established peptide databases. This exopeptidase‐based strategy is currently under development and was not experimentally evaluated in the present study.

We note that changing the enzyme from trypsin to an exonuclease (X‐ToF) can provide the ability of this mixed‐scale fluidic platform to sequence nucleic acids, such as DNAs and RNAs. Because the exonuclease is a processive enzyme, the loading of the DNA or RNA is achieved using the input/output channels shown in Figure 1. Thus, by simple changeover of the enzyme with no architectural changes, the P‐ToF/X‐ToF chips can be programmed to process biopolymers and elucidate their monomer content to provide seamless multi‐omics data.

## 4 Experimental Section

### 4.1 Materials and Reagents

All chemicals and reagents were purchased from commercial suppliers and used as received without further purification. Affinity‐purified Amyloid‐β (Aβ1‐40; DAEFRHESGYEVHHQKLVFFAEDVGSNKGAIIGLMVGGVV) and its four peptide fragments, N5 (DAEFR), HisRich (HESGYEVHHQK), HydroCore (LVFFAEDVGSNK), and HydroTail (GAIIGLMVGGVV) were obtained from the Synthetic Chemical Biology Core Facility (SCB Core) at the University of Kansas. Trypsin (from bovine pancreas) was purchased from Sigma‐Aldrich (St. Louis, MO, USA). AZ 9260 positive photoresist and AZ 300 MIF developer were obtained from MicroChemicals GmbH (Ulm, Germany). Silicon/silicon dioxide (Si/SiO_2_) substrates and <100> Si wafers were purchased from University Wafer, Inc. (South Boston, MA, USA). Buffered oxide etchant (BOE, 10:1) was sourced from Sigma‐Aldrich (St. Louis, MO, USA). The UV‐curable formulation was comprised of 97 wt% MD700 (Solvay Solexis, Italy) and 3 wt% Darocur 1173 photoinitiator (Sigma‐Aldrich, St. Louis, MO, USA). NOA72 optical adhesive was supplied by Norland Products (Jamesburg, NJ, USA). Poly (ethylene terephthalate; PET) films (250 µm thickness) were acquired from Goodfellow (Coraopolis, PA, USA). Cyclic olefin polymer (COP, Type 1060R) sheets were secured from STRATEC SE (Birkenfeld, Germany), and cyclic olefin copolymer (COC, Type 8007) was obtained from TOPAS Advanced Polymers (Florence, KY, USA). Phosphate‐buffered saline (PBS) was purchased from Sigma‐Aldrich (St. Louis, MO, USA), and molecular biology‐grade water was obtained from Thermo Fisher Scientific (Waltham, MA, USA).

### 4.2 Fabrication of the Silicon Master

Micro‐ and nanoscale features were first defined on a silicon (Si) wafer using a combination of optical lithography and focused ion‐beam (FIB) milling (see Figure 6). For microscale patterning, a thermally oxidized Si wafer (300 nm SiO_2_) was coated with a positive photoresist (AZ 9260) by spin coating at 4000 rpm for 60 s, followed by a soft bake at 100°C for 5 min. Microchannel patterns were transferred from a chrome photomask by UV exposure and subsequently developed using AZ 300 MIF developer. After pattern development, the exposed oxide layer was removed using BOE (10:1), and the underlying Si was etched (wet or dry etching, depending on feature geometry) to produce microstructures with depths of approximately 4–8 µm. Nanoscale elements, including funnels, nanochannels, flight tubes, and nanopores were created by FIB milling at 30 kV accelerating voltage. Beam currents were adjusted between 10–300 pA depending on the desired feature dimensions, with bitmap milling employed for precise pattern definition in three dimensions. This hybrid lithography/FIB approach enabled seamless integration of micro‐ and nanofluidic elements within a single Si master [37].

**FIGURE 6:**
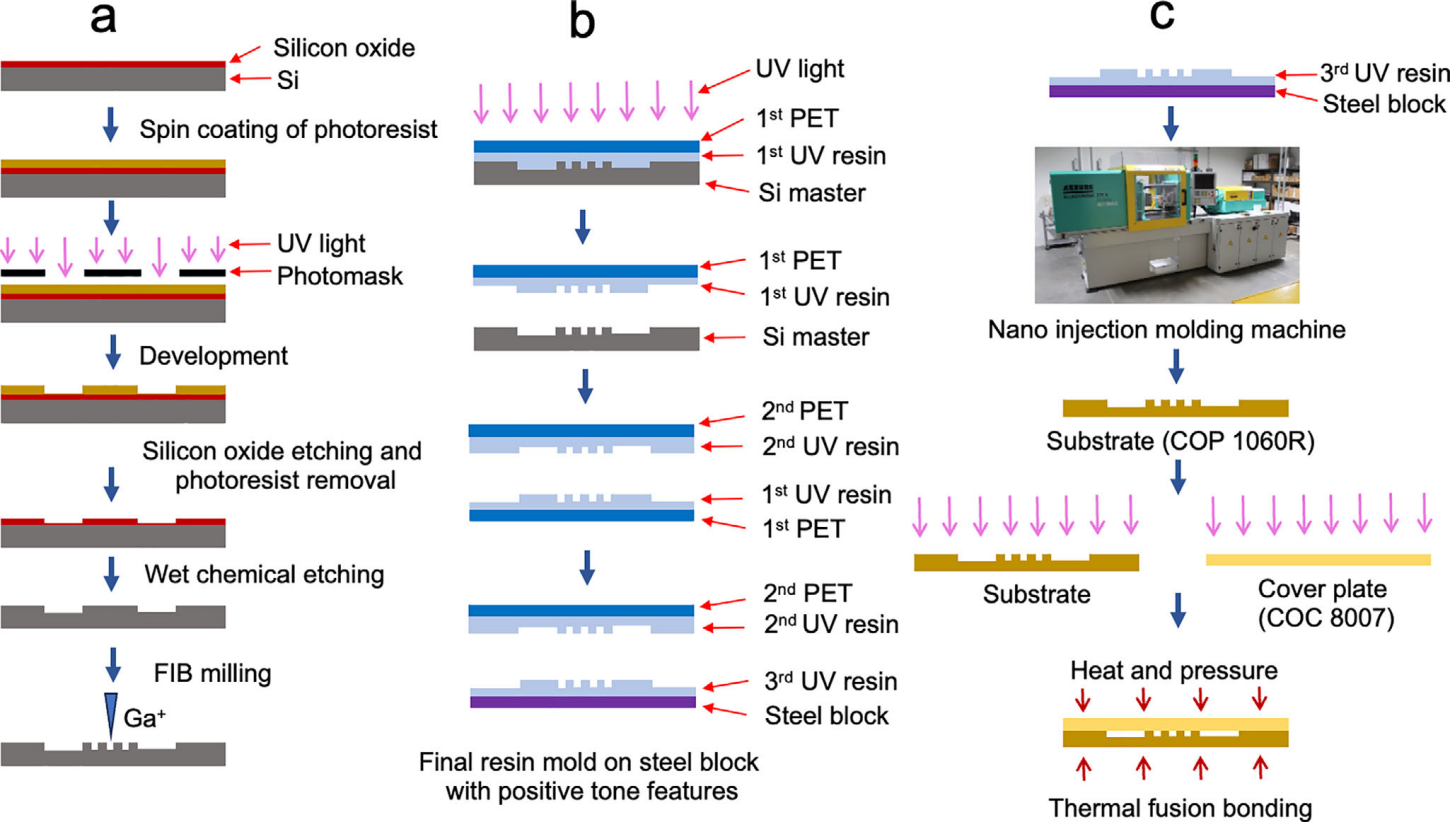
P‐ToF chip fabrication process. (a) Illustration of the Si master fabrication process, which includes microstructuring through photolithography and wet‐chemical etching, followed by nanostructuring with FIB milling using a FEI Quanta 3D FEG FIB/SEM employing Ga+ ions. Specifically, the funnel structures were patterned at 30 kV with a current of 300 pA, while the flight tube and nanopores were milled at 30 kV with a current of 10 pA in a bitmap mode. (b) Production of a UV resin mold by transferring the Si master pattern onto a metal block. (c) Formation of the COP substrate via injection molding with the resin mold, followed by UV/O_3_ treatment and thermal fusion bonding of the COC cover plate to the COP substrate. Abbreviations: COP = cyclic olefin polymer, COC = cyclic olefin copolymer, PET = polyethylene terephthalate. MD 700 was used as the UV resin.

### 4.3 Replication of Resin Mold Inserts

To preserve the Si master and enable scalable replication, features were transferred into a fluorinated UV‐curable resin (MD700) containing 3 wt% Darocure 1173 photoinitiator. A small volume of the resin was dispensed onto the Si master surface, and a flexible polyethylene terephthalate (PET) film, serving as a mechanical support, was gently placed on top to spread the resin uniformly. Polymerization was achieved by UV irradiation (365 nm), producing a first‐generation resin replica with positive‐tone features. To avoid repeated use of the Si master, two sequential replication steps were performed to generate intermediate molds, ultimately yielding a final resin mold insert with positive‐tone structures for mixed‐scale injection molding.

For injection molding compatibility, the final resin replica was transferred onto a stainless‐steel block. Prior to resin casting, the metal surface was mechanically polished, treated with O_2_ plasma to enhance adhesion, and coated with a thin NOA72 adhesive layer. UV curing produced a mechanically stable mold insert suitable for thermoplastic replication. Although direct replication from the Si master to the steel block is possible, intermediate replication steps were implemented to minimize mechanical stress and prolong master lifetime due to the complexity of its fabrication (i.e., combination of photolithography and FIB milling) [63].

### 4.4 Injection Molding of Thermoplastic Substrates

Nanofluidic substrates were fabricated by injection molding using an all‐electric injection molding system (Arburg 307A, Rocky Hill, CT) equipped with a master unit die (MUD). The resin mold inserts were mounted in the stationary half of the die and defined the micro/nanostructures, while a polished blank formed the opposing cavity surface. COP pellets were melted at elevated temperature (nozzle temperature ∼270°C) and injected into the mold cavity maintained at ∼80°C. After filling, a holding pressure (∼800 bar) was applied briefly to compensate for shrinkage, followed by cooling through conductive heat transfer to the mold insert and blank. Devices were demolded after a cooling period of ∼60 s. This process enabled rapid cycle times (<1 min per device) and reproducible replication of nanoscale features, as verified by SEM and AFM characterization. Multiple resin inserts could be generated from a single Si master, and each insert supported fabrication of >100 polymer devices without significant degradation of feature fidelity.

### 4.5 Device Assembly Using Thermal Fusion Bonding

The molded COP substrates were sealed with cyclic olefin copolymer (COC 8007) cover plates using a hybrid thermal fusion bonding strategy that exploits the difference in glass transition temperatures (T_g_) between the two materials (COP ∼98.8°C; COC ∼78°C). Prior to bonding, reservoirs were drilled into the COP substrate. Both substrate and cover plate were treated with UV/O_3_ irradiation (21.4 mW/cm^2^ for 6 min) to reduce surface hydrophobicity and promote interfacial adhesion during thermal fusion bonding. The Aβ concentrations used in this study (1 nM and 100 pM) were substantially below the reported critical aggregation concentration of approximately 90 nM for Aβ42, with negligible aggregation reported below 20 nM under the conditions investigated by Novo et al. Accordingly, concentration‐dependent Aβ self‐aggregation is not expected to be significant under the experimental concentrations used here [64]. Longer activation times were not used to minimize the extent of surface charge generation that can affect the molecular capture rate by the in‐plane nanopores [65]. Bonding was performed under controlled temperature and pressure resulting in enclosed micro/nanofluidic networks with high process yield (>90%) and strong interfacial integrity [66].

### 4.6 Atomic Force Microscopy

High‐resolution AFM scans (7.5 µm × 7.5 µm, 256 × 256 pixels, scan rate 0.5 Hz) were secured using an SPM HT‐9700 (Shimadzu Scientific Instruments, Columbia, MO) tapping mode AFM with super sharp Si tips (curvature radius of 2 nm, Nanosensors AG, Neuchatel, Switzerland). The dimensions of nanochannels, in‐plane nanopores and height of the surface immobilized enzyme layer were determined using AFM imaging.

### 4.7 Scanning Electron Microscopy

SEM images were acquired using an FEI VERSA 3D Dualbeam field emission/low vacuum SEM instrument. A 2 nm‐thin conductive iridium layer was sputter‐coated onto the devices using an EMS 150ES sputter coater before SEM imaging. All images were acquired using a 5.0 kV accelerating voltage and 8.7 mm working distance. The SEM images of the Si mold masters were collected using a Quanta 3D DualBeam FEI FIB‐SEM instrument and were analyzed using the instrument's software and ImageJ.

### 4.8 COMSOL Simulations

Finite element simulations were conducted using COMSOL Multiphysics v5.5 (COMSOL Inc.) to model the electric potential distribution and resulting electric field strengths throughout the fluidic network of the P‐ToF chip.

### 4.9 Data Analysis

Current traces acquired from the transimpedance amplifier (TIA) were processed using MATLAB R2024a (MathWorks, Inc.) with a custom‐built analysis program (EventFinder v2.55). Signals were digitally filtered using a 10 kHz low‐pass filter and a 100 Hz high‐pass filter prior to event detection. Event thresholds were defined relative to the background noise level and set at approximately three times the standard deviation (3σ) of the baseline root‐mean‐square (RMS) current noise. Translocation events were identified when the current amplitude exceeded 3σ of the baseline noise. For each detected event, key parameters including ΔI (pore 1), ΔI (pore 2), dwell time (t_D_) at pore 1 and pore 2, and time‐of‐flight (ToF) were extracted from the EventFinder output. To minimize variability arising from minor pore‐to‐pore dimensional differences and device‐dependent fluctuations, ΔI and t_D_ values from both pores were averaged prior to statistical analysis in some cases; not averaged for the machine learning phases of the data analysis. Current blockades were normalized to the open‐pore current (ΔI/I_0_) for box plot generation, principal component analysis (PCA), and subsequent machine learning workflows. Box plots were generated using OriginPro 2024b (OriginLab Corp.), and pie charts were prepared in Microsoft Excel.

### 4.10 Machine Learning

Model training and evaluation were performed in Python using the scikit‐learn (sklearn) library. Linear discriminant analysis (LDA), quadratic discriminant analysis (QDA), support vector machine (SVM), multi‐layer perceptron (MLP), and Random Forest (RF) classifiers were evaluated with the RPS data generated in this study. Matplotlib was used for visualization. Input features were standardized using StandardScaler for LDA, QDA, SVM, and MLP models. An 80/20 stratified train‐test split was applied. Model performance was further assessed using 5‐fold stratified cross‐validation to preserve class balance. The RF classifier was constructed using 500 decision trees (n_estimators = 500), random_state = 0, and n_jobs = −1, with all other hyperparameters left as default values. The MLP classifier consisted of two hidden layers of 50 neurons each (hidden_layer_sizes = (50, 50)), using the rectified linear unit (ReLU) activation function and the Adam optimizer (alpha = 1×10^−4^, learning_rate_init = 1×10^−3^, max_iter = 2000, early_stopping = False). For reproducibility, all models were initialized with RANDOM_STATE = 0. Performance metrics included accuracy and macro‐F1 score. Feature importance for the RF model was evaluated using both Gini importance (training‐derived) and permutation importance on the held‐out test set (*n*_repeats_ = 50).

## Author Contributions

**Suresh Shivanka**: conceptualization, investigation, Writing – original draft, formal analysis. **Randy F. Espinal Cabrera**: formal analysis, writing – original draft. **Maximillian Chibuike**: investigation. **Pramodi Vithanage**: investigation. **Indu A. Chandrasoma**: investigation. **Pubudu Premarathne**: investigation. **Matthew Verber**: formal analysis. **Steven A. Soper**: conceptualization, funding acquisition, writing – review and editing, supervision, resources.

## Conflicts of Interest

The authors declare no conflicts of interest.

## Supporting information




**Supporting File**: smtd71044‐sup‐0001‐SuppMat.docx.

## Data Availability

The data that support the findings of this study are available on request from the corresponding author. The data are not publicly available due to privacy or ethical restrictions.
