# Supplementary material for: Peptide Fingerprinting of Single Protein Molecules Using In‐Plane Nanopore Sensors Integrated to an Extended Immobilized Nanoscale Enzymatic Reactor (eINER)
Source: Small Methods. 2026 Sep 16;10(20):e71044. doi: 10.1002/smtd.71044 (PMC13706642; doi:10.1002/smtd.71044)
Supplement: Supplementary file 1 [file SMTD-10-e71044-s001.docx]

**Supporting Information**

**Peptide Fingerprinting of Single Protein Molecules Using In-Plane Nanopore Sensors Integrated to an Extended Immobilized Nanoscale Enzymatic Reactor (eINER)**

Suresh Shivanka,^1,2^ Randy F. Espinal Cabrera,^1,2^ Maximillian Chibuike,^1,2^ Pramodi Vithanage,^1,2^ Indu A. Chandrasoma,^1,2^ Pubudu Premarathne,^1,2^ Matthew Verber,^2,3^ and Steven A. Soper^1,2,4,5,6*^

^1^Department of Chemistry, The University of Kansas, Lawrence, KS 66045

^2^Center of BioModular Multiscale Systems for Precision Medicine

^3^University of North Carolina, Chapel Hill, NC

^4^Department of Mechanical Engineering, The University of Kansas, Lawrence, KS 66045

^5^Bioengineering Program, The University of Kansas, Lawrence, KS 66045

^6^KU Cancer Center, University of Kansas Medical Center, Kansas City, KS 66160

**Figure S1.** Geometry and transport pathways of the eINER–DNP-ToF components of the P-ToF chip with cross-sectional features used for voltage-division analysis. (**a**) Geometry of the eINER and DNP-ToF reader. Schematic representation of the extended immobilized nanoscale enzymatic reactor (eINER) integrated with the sensing channel containing a dual in-plane nanopore time-of-flight (ToF) reader. The reactor consists of two enzyme input nanochannels (250 nm × 250 nm cross-section) that converge into a curved central reaction chamber, approximated as 0.8 of an annulus (inner radius r_i_ =250 nm; outer radius r_o_ = 500 nm). The curved region connects to a vertically aligned sensing nanochannel containing two in-plane nanopores separated by a defined flight length (flight tube). (**b**) Molecular transport pathway inside the P-ToF chip during eINER digestion. Molecules travel through the eINER under electroosmotic flow (EOF) from the anode ((+) electrode) toward the cathode (ground electrode). Accordingly, the effective reaction length is defined as one enzyme input channel plus half of the curved channel corresponding to 7.50 µm + 0.94 µm = 8.44 µm (i.e., half of the total eINER length). Following enzymatic digestion of Aβ, the resulting peptide fragments are transported into the downstream sensing channel for detection and identification. The schematic is not drawn to scale. (**c**) Cross-sectional geometries used for voltage-division analysis to determine the electric field strength. Schematic cross-sections of the segmented molecular transport pathway (1.0 V applied), illustrating the transition from 250 × 250 nm² enzymatic regions to 50 × 50 nm² sensing channels and 10 nm nanopore constrictions where electric-field localization is concentrated.

### 1 Nanoscale Transport and Electric Field Hierarchy

Once Aβ was introduced into the sample input reservoir of P-ToF, it was transported through the microchannel network and entered the appropriate nanochannels by electrokinetic control (**Figure S1a**). A positive voltage was applied at the sample input reservoir, and the circuit was completed by grounding at the output end of the DNP-ToF reader.

The dimensions of the eINER and DNP-ToF reader were selected to establish a hierarchical electric-field distribution across the device, as guided by electrical modeling and COMSOL simulations [1]. The larger 250 × 250 nm² eINER channels maintain a relatively low electric field, whereas the reduced cross-section of the DNP-ToF reader (50 × 50 nm² channels and ~10 nm nanopores) produces higher local electric fields. This field gradient facilitates electrokinetic extraction and capture of peptide products generated within the eINER into the downstream DNP-ToF reader for single-molecule detection. The fabrication of our devices was performed exclusively from nano-injection molding using a resin stamp, which produces an inter-device structural variation of <5% relative standard deviation [2].

### 2 eINER Geometry and Transport Parameters

The eINER consists of quasi-square nanochannels with width (w) and height (d) of 250 nm and in our case, w = d. For digestion prior to sensing, the effective reaction length was defined as (l) (**Figure S1**), where $l = 8.44\times{10}^{-6} m$. Electrokinetic simulations under a 1.0 V bias yielded an average velocity $\left( v \right) \mathrm{of} 1.5\times{10}^{-3}m s^{-1}$ for Aβ. The diffusion coefficient of monomeric Aβ was taken as (D_A_) $= 1.0\times{10}^{-10} m^{2}s^{-1}$.

### 3 Sampling Volume of the eINER

The total sampling volume of the eINER was calculated from the two straight enzyme input channels and the central curved annular segment (see **Figure S1**). The rectangular channel cross-sectional area is $A = 6.25\times{10}^{4} nm^{2}$. The total reactor volume is given by:

$V_{total}= 2AL + 0.8\pi\left( r_{o}^{2}- r_{i}^{2} \right)d$	(S1)

where V_total_ is the total eINER volume; A is the rectangular cross-sectional area; L is the length of one straight enzyme input channel (7.5 µm); r_o_ is the outer radius of the curved channel (500 nm); r_i_ is the inner radius of the curved channel (250 nm); d is the channel depth (250 nm); and 0.8 represents the fraction of the full annulus corresponding to the curved segment (**Figure S1a**).

Substituting the geometrical parameters gives $V_{total}= 1.0553 \mu m^{3}\approx1.06 fL$.

### 4 Voltage Division and Electric-Field Distribution Along the Biased Transport Pathway

Each nanofluidic segment along the voltage biased pathway was modeled as an ohmic resistor under the assumptions of uniform electrolyte conductivity and negligible surface conduction. The electrical resistance of segment *i* scales geometrically as:

$R_{i}\propto\frac{L_{i}}{A_{i}}$	(S2)

where L_i_ is the longitudinal length and A_i_ is the cross-sectional area of segment *i*. For a total applied bias V_total_, the voltage drop across segment i is given by:

$\Delta V_{i}=\frac{V_{total}\left( \frac{L_{i}}{A_{i}} \right)}{\sum_{j} \left( \frac{L_{j}}{A_{j}} \right)}$	(S3)

In this expression, L_j_ and A_j_ denote the length and cross-sectional area of every segment j included in the nanoscale transport pathway between the electrodes (**Figure S1**). The summation ∑_j_(L_j_/A_j_) therefore represents the total geometric resistance of all nanofluidic elements in series, including the enzyme input channel, curved INER segment, narrow sensing-channel sections, dual in-plane nanopores, and post-pore region. Microchannels and funnels were excluded from the summation because their much larger cross-sectional areas yield negligible resistance and thus, negligible voltage drop relative to the nanoscale sections. The average electric field in each segment was calculated as:

$E_{i}=\frac{\Delta V_{i}}{L_{i}}$	(S4)

with conversion to V cm^-1^ performed using 1 μm = 10^−4^ cm. **Table S1** shows the geometrical values used herein for the P-ToF chip and the resulting electric field in each segment of the analyte transport pathway.

**Table S1.** Voltage division and average electric-field strengths across the biased nanoscale transport pathway (1.0 V applied). Each segment was modeled as an ohmic resistor with resistance scaling as L/A under uniform electrolyte conductivity. Most of the applied voltage drop is localized within the 50 × 50 nm² sensing channel and nanopore constrictions, while the 250 × 250 nm² enzymatic regions remain in a lower electric field regime.

| **Section** | **Length (µm)** | **Cross-section (nm × nm)** | **ΔV (V)** | **E (V cm^-1^)** |
| --- | --- | --- | --- | --- |
| Enzyme input channel | 7.50 | 250 × 250 | 0.0250 | 33.4 |
| Half curved channel | 0.94 | 250 × 250 | 0.00314 | 33.4 |
| Pre-1st pore section | 1.00 | 50 × 50 | 0.0835 | 835 |
| In-plane nanopore 1 | 0.01 | 10 × 10 | 0.0209 | 2.09 × 10⁴ |
| Flight tube | 10.0 | 50 × 50 | 0.8349 | 835 |
| In-plane nanopore 2 | 0.01 | 10 × 10 | 0.0209 | 2.09 × 10⁴ |
| Post-2nd pore section | 3.50 | 250 × 250 | 0.0117 | 33.4 |

#### 4.1 Quantitative Interpretation

The voltage division reveals three distinct electric-field regimes along the transport pathway (see **Table S1**). In the 250 × 250 nm² eINER regions, the average electric field was approximately 33 V cm^−1^. These wider nanochannels exhibited relatively low fluidic resistance and therefore experienced only a small fraction of the voltage drop across the fluidic pathway. As a result, the enzymatic reactor operates under comparatively small voltage drops preserving a stable microenvironment for immobilized trypsin.

In contrast, the 50 × 50 nm² sensing-channel segments in the DNP-ToF reader support an intermediate electric field of approximately 835 V cm^−1^. Because resistance scales inversely with cross-sectional area, these narrower sections coupled with the relatively long length produce the majority of the voltage drop within the fluidic circuit. The flight tube between the two nanopores therefore establishes the dominant electrophoretic and EOF driving forces responsible for single-molecule transport toward the cathodic reservoir, which in this case is the reservoir at the terminal end of the DNP-ToF reader.

The highest electric field was localized within the 10 nm in-plane nanopores, where fields reach approximately 2 × 10^4^ V cm^−1^. Although each nanopore drops only ~0.021 V, the extremely short length (0.01 µm) results in a small voltage drop relative to the 50 × 50 nm flight tube. This strong localization is essential for sensitive current blockage detection at the dual nanopore reader while remaining spatially confined to the nanopore.

### 5 Peak Detection and Peak-Pair Assignment

RPS peaks were identified from samples in the current trace that exceeded an amplitude threshold defined as approximately three times the standard deviation (3σ) of the baseline RMS open pore current noise. A 1 ms blanking interval following the first threshold crossing was used to prevent double counting of a single peak. For each detected peak, the maximum amplitude was determined until the signal returned to baseline, with the most negative amplitude used for negative-going peaks. The peak dwell time (t_D_) was determined as the full width at half maximum (FWHM) relative to the maximum-amplitude sample. Peaks with dwell times outside the predefined range of 0.1–1 ms were excluded.

Following peak identification, peak pairs corresponding to sequential translocation through the first and second in-plane nanopores in series were assigned based on their temporal separation. The time-of-flight (ToF) was calculated as the time between the maximum-amplitude samples of the first and second RPS peaks. Peak pairs with ToF values outside the predefined range of 1–60 ms were excluded. Peak-pair assignments were visually verified using annotated current traces identifying the first peak, second peak, and unpaired peaks.

**Figure S2.** Structural and mechanistic representation of trypsin catalysis. (a) Ribbon structure of trypsin highlighting the overall tertiary fold. Enlarged view shows the catalytic triad residues His57, Asp102, and Ser195, along with Asp189 located at the base of the S1 specificity pocket. The S1 specificity pocket is formed by loops comprising residues 189–195, 214–220, and 225–228. The catalytic triad forms a charge-relay network that activates Ser195 for nucleophilic attack, while Asp189 confers substrate specificity for lysine and arginine residues. (b) Stepwise serine protease catalytic mechanism illustrating formation of the first tetrahedral oxyanion intermediate, collapse to the acyl–enzyme intermediate, water-mediated deacylation through a second tetrahedral intermediate, and release of the cleaved peptide product.

### 6 Catalytic Mechanism and Structural Accessibility of Immobilized Trypsin in the eINER

Trypsin is a serine protease that catalyzes peptide-bond hydrolysis at the C-terminus of lysine (Lys)/arginine (Arg) residues. Unlike processive enzymes, trypsin operates through a distributive mechanism requiring an independent binding event for each cleavage reaction. Catalysis occurs within a solvent-accessible surface cleft that houses the canonical serine protease catalytic triad consisting of Ser195, His57, and Asp102 (chymotrypsin numbering; **Figure S2a**) [3].

The catalytic triad operates through a charge-relay network that activates Ser195 for nucleophilic attack. Asp102 stabilizes and orients His57 increasing its basicity. Upon substrate binding, His57 abstracts the proton from the hydroxyl group of Ser195 generating a nucleophilic alkoxide. The activated Ser195 attacks the carbonyl carbon of the scissile peptide bond forming the first tetrahedral oxyanion intermediate, which is stabilized by backbone amide hydrogen bonds within the oxyanion hole. Collapse of this intermediate results in cleavage of the peptide bond, formation of the acyl-enzyme intermediate, and release of the N-terminal fragment. In the deacylation step, a water molecule enters the active site and is deprotonated by His57, generating a hydroxide nucleophile. This hydroxide attacks the carbonyl carbon of the acyl-enzyme forming a second tetrahedral intermediate that is stabilized by the oxyanion hole. Collapse of this intermediate breaks the ester linkage to Ser195, regenerates the free enzyme, and releases the C-terminal fragment (**Figure S2b**) [3].

Substrate specificity is governed by the S1 specificity pocket, which contains Asp189 at its base. The negatively charged carboxylate of Asp189 forms a stabilizing electrostatic interaction with the positively charged ε-amino group of Lys or the guanidinium group of Arg. This interaction properly orients the peptide backbone such that the scissile bond is precisely positioned relative to Ser195. Thus, cleavage selectivity arises from cooperative engagement between the S1 pocket (substrate recognition) and the catalytic triad (chemical transformation). Crystallographic analysis indicates that trypsin is a compact globular protein of 4–5 nm in diameter. Importantly, the active site resides within a surface-exposed cleft rather than being buried within the protein core. The S1 pocket is recessed but remains solvent accessible. This structural topology is critical for maintaining enzymatic function following covalent immobilization to the eINER surface.

Trypsin contains ~14 lysine residues distributed predominantly across solvent-exposed surface loops [4]. These residues serve as reactive handles during covalent immobilization onto carboxyl-functionalized COP/COC substrates via EDC/NHS coupling chemistry (**Figure S3a**). UV/O₃ activation generates surface-confined −COOH groups that react with primary amines of lysine residues to form stable amide linkages. Structural mapping shows that only a small subset of lysine residues are located proximal to the catalytic cleft, whereas most are positioned distal from both the catalytic triad and the S1 pocket [5]. This spatial separation reduces the likelihood that covalent attachment sterically occludes substrate access to the catalytic site or perturbs the catalytic geometry. Because the active site is solvent-exposed and the S1 pocket remains accessible after surface tethering, immobilized trypsin within the eINER retains catalytic competence and substrate selectivity. Surface characterization after immobilization using AFM revealed an average enzyme height of 4.76 ± 0.36 nm, consistent with literature values for trypsin in its catalytically competent orientation (**Figure S3b-d**).

COMSOL simulations showed that the electric field is focused within the eINER chamber during enzyme loading, generating localized electrokinetic transport in that region while minimizing flow into adjacent channels. This field confinement enables site-specific immobilization of trypsin nearly exclusively within the eINER and minimizes enzyme leakage into surrounding micro- and nanochannels (**Figure S3e**). Consequently, the observed near-quantitative digestion efficiency in the nanofluidic reactor is consistent with preserved accessibility of both the catalytic triad and the S1 specificity pocket following covalent immobilization.

**Figure S3**. Enzyme immobilization process onto the eINER. (**a**) EDC/NHS-mediated coupling mechanism for covalent immobilization of trypsin onto a carboxyl-functionalized COP/COC surface. Surface carboxyl groups (–COOH) are first activated by EDC to form a reactive O-acylisourea intermediate, which is subsequently stabilized by N-hydroxysuccinimide (NHS) to generate a surface-bound NHS ester. The ε-amino group (–NH₂) of lysine residues on trypsin then performs nucleophilic attack on the activated ester carbonyl forming a tetrahedral intermediate that collapses with displacement of NHS yielding a stable amide (–CONH–) bond. This reaction results in covalent coupling of trypsin to the COP/COC thermoplastic surfaces via the lysine side chains. (**b**) Atomic force microscopy (AFM) image of the enzyme immobilized surface. (**c**) AFM height profile of the trypsin-immobilized surface. AFM line-scan analysis showing the height profile of the trypsin-functionalized surface. The cross-sectional profile indicates a measured feature height of approximately 4.79 nm consistent with the presence of immobilized trypsin molecules on the substrate. (**d**) Histogram of immobilized trypsin height for a total of 50 counts showing the average height was 4.76 ± 0.36 nm. (**e**) COMSOL simulation illustrating site-specific electrokinetic confinement during trypsin immobilization. Finite element simulations (COMSOL Multiphysics) showing the velocity magnitude distribution (1 × 10^-3^ m/s) at applied voltages of 1.0 V, 2.0 V, and 5.0 V. The electric field is focused within the circular eINER chamber, generating localized electrokinetic transport within this region while minimizing flow into adjacent channels. This field confinement enables site-specific immobilization of trypsin within the eINER and minimizes enzyme leakage into surrounding channels.

Cleavage at the Arg5, Lys16, and Lys28 sites produces four peptide fragments upon complete digestion of Aβ within the eINER. In addition, five partially digested peptide fragments could also be produced (**Table S2**). The digestion process continues throughout the enzyme input channel and the half-curved channel after which all digestion products were transported into the DNP-ToF reader. This transport route defines the molecular pathway through the device (**Figure S1b, c**) for the sample. In the experiments reported herein, the input and output channels and their associated in-plane nanopores were not used to extend the residence time of Aβ within the eINER for more complete digestion under these conditions. Therefore, no RPS signals were generated to indicate entry of a single Aβ molecule into the eINER.

Also shown in **Table S2** are the calculated apparent electrophoretic mobility (µ_app_) for the trypsin peptide fragments produced in the eINER for the enzymatic action on Aβ, which were calculated from their ToF values (see **Figure 3** in the main text). The µ_app_ was calculated using (l/(E × ToF)), where l is the length of the flight tube (10 µm) and E is the electric field strength in the flight tube (835 V/cm). Because the net charge on HydroTail is 0.0, it moves at the same speed as the EOF.

**Table S2.** Physicochemical properties of Aβ and its trypsin enzymatic digestion fragments. Molecular weight, pI, net charge, and GRAVY values were calculated using the ExPASy ProtParam tool based on primary sequence. Hydrodynamic radii were estimated using an empirical scaling relationship for intrinsically disordered peptides (Rh ≈ 0.22 × N0.57 nm, where N is the number of residues). The apparent electrophoretic mobilities were calculated from the measured ToF for each trypsin peptide fragment generated from Aβ. NA – these are potential peptide fragments that could be generated from Aβ for partial digestion. In this case, the ToF were not measured.

| **Name** | **Amino acid sequence** | **Mass (g/mol)** | **pI** | **Rh (nm)** | **Net charge** | **GRAVY**  **Scores** | **Apparent Electrophoretic Mobility (cm^2^/Vs)** |
| --- | --- | --- | --- | --- | --- | --- | --- |
| **Intact – Undigested** | | | | | | |  |
| Aβ (40) | DAEFRHESGYEVHHQKLVFFAEDVGSNKGAIIGLMVGGVV | 4343.81 | 5.25 | 1.80 | -2.9 | 0.06 | 0.26 × 10^-4^ |
| **Peptide fragments - Complete digestion** | | | | | | |  |
| N5 (5) | DAEFR | 636.12 | 4.17 | 0.55 | -1.3 | -1.38 | 0.65 × 10^-4^ |
| HisRich (11) | HESGYEVHHQK | 1350.08 | 6.10 | 1.21 | -0.9 | -2.02 | 0.32 × 10^-4^ |
| HydroCore (12) | LVFFAEDVGSNK | 1325.46 | 4.07 | 0.97 | -1.0 | 0.33 | 0.57 × 10^-4^ |
| HydroTail (12) | GAIIGLMVGGVV | 1085.36 | 5.52 | 0.91 | 0.0 | 2.29 | 1.04 × 10^-4^ |
| **Peptide fragments - Partial digestion** | | | | | | |  |
| Fragment 1 (16) | DAEFRHESGYEVHHQK | 1969.03 | 5.84 | 1.03 | -2.1 | -1.23 | NA |
| Fragment 2 (23) | HESGYEVHHQKLVFFAEDVGSNK | 2733.23 | 5.52 | 1.28 | -2.0 | -0.74 | NA |
| Fragment 3 (24) | LVFFAEDVGSNKGAIIGLMVGGVV | 2410.30 | 4.95 | 1.31 | -1.0 | 0.85 | NA |
| Fragment 4 (28) | DAEFRHESGYEVHHQKLVFFAEDVGSNK | 3294.65 | 5.42 | 1.43 | -3.0 | -0.55 | NA |
| Fragment 5 (35) | HESGYEVHHQKLVFFAEDVGSNKGAIIGLMVGGVV | 4038.81 | 5.31 | 1.63 | -2.0 | 0.21 | NA |

**7 Peclet Analysis of Molecular Transport in the eINER**

To evaluate the relative contributions of convective and diffusive contributions to molecular transport within the eINER, the Peclet number (Pe) was calculated. The Peclet number describes the ratio of convective transport to diffusive transport and is defined as;

$Pe=\frac{\mathrm{vL}}{D_{A}}$	(S5)

where v is the average linear velocity of the fluid, L is the characteristic transport length, and D_A_ is the diffusion coefficient of the analyte, in this case Aβ. Substituting values for the current set of experiments gives Pe ≈ 1.27 x 10^2^.

A Peclet number significantly greater than unity indicates that transport within the reactor is dominated by convective flow, which in this system arises from the EOF. Consequently, peptides are transported primarily in the longitudinal direction by EOF rather than by molecular diffusion.

Although axial transport is convection-dominated, diffusion remains important in the transverse direction, where peptides must diffuse from the bulk flow toward the enzyme-functionalized channel wall to undergo proteolytic cleavage. Because the nanochannel cross-section is only 250 nm × 250 nm, the transverse diffusional distance is small enabling rapid molecular exchange between the bulk flow and the enzyme-decorated surface. This transport regime allows efficient delivery of substrate molecules to immobilized trypsin. The Peclet number therefore supports the transport model used for the eINER, where EOF governs longitudinal peptide transport while diffusion enables access to the enzyme-coated surfaces required for digestion.

### 8 Damköhler Analysis for Surface Immobilized Trypsin Digestion

To assess whether digestion of Aβ by immobilized trypsin within the eINER is limited by enzymatic reaction kinetics or by diffusive transport of substrate to the channel walls, a surface-reaction Damköhler number (Da) was evaluated. Da compares the characteristic rate of substrate consumption at a reactive surface to the rate of diffusive replenishment from the bulk and is commonly used in micro- and nanofluidic wall-reaction analyses. For a first-order wall reaction in a channel of characteristic height H, Da can be expressed as;

$Da=\frac{k_{s}H}{D_{A}}$(S6)

where k_s_ is an effective surface reaction rate constant (m s⁻¹) and D_A_ is the diffusion coefficient of the substrate in solution, which in this case is Aβ.

For an immobilized enzyme obeying Michaelis-Menten kinetics under dilute-substrate conditions ([S] ≪ K_m_), the surface reaction rate constant can be approximated as;

$k_{s}\approx\left( \frac{k_{cat}}{K_{m}} \right)\left[ B_{max} \right]$ (S7)

where k_cat_ is the catalytic turnover number, K_m_ is the Michaelis constant, and [B_max_] is the surface density of immobilized enzyme active sites (mol m^-2^). This formulation has been used extensively to describe enzymatic capture and consumption at reactive microchannel walls. Substituting Eq. (S7) into Eq. (S6) yields;

$Da=\frac{\left( \frac{k_{cat}}{K_{m}} \right)\left[ B_{max} \right]H}{D}$	(S8)

The nanoscale enzymatic reactor consists of square nanochannels with a cross-section of 250 nm × 250 nm as noted above, giving a characteristic channel height of 250 nm.

The diffusion coefficient of monomeric Aβ under high-ionic-strength conditions was taken as $1.0\times10^{-10} m^{2}s^{-1}$, which is consistent with reported diffusion coefficients for small peptides and unfolded proteins in aqueous buffers.

From the surface density of immobilized trypsin,

$\left[ B_{max} \right]=\frac{N}{A}=\frac{274}{16.885}=16.22 molecules \mu m^{-2}$	(S9)

and using Avogadro’s number, $N_{A}=6.022\times{10}^{23} molecules mol^{-1}$

$\left[ B_{max} \right]=\frac{1.622\times10^{13}}{6.022\times10^{23}}=2.69\times10^{-11} mol m^{-2}$	(S10)

#### 8.1 Trypsin Kinetic Parameters

Direct kinetic constants for immobilized trypsin are not available in the literature. Therefore, representative solution-phase trypsin kinetics for protein/peptide substrates were used to establish an order-of-magnitude scaling, as is standard practice in Damköhler analyses of enzymatic microreactors. Reported kinetic values for bovine trypsin with peptide substrates indicate $K_{m}$ ≈ 6–10 µM and thus, a catalytic efficiency of $\frac{k_{cat}}{K_{m}}$ ≈ 10^6^–10^7^ M^-1^ s^-1^ for peptide substrates cleaved at lysine or arginine residues [6, 7]. These values correspond to turnover numbers, $k_{cat}$, in the range of 10–100 s^-1^, which are consistent with classical trypsin kinetics.

#### 8.2 Damköhler Number Calculation

Using Eq. (S8) with the parameters above $Da = \left( 6.72\times10^{-8} \right)\left( \frac{k_{cat}}{K_{m}} \right)$. Using the literature range for trypsin $\frac{k_{cat}}{K_{m}}= 1\times10^{3}m^{3} mol^{-1} s^{-1}$ to $1\times10^{4} m^{3} mol^{-1} s^{-1},$ under the best-case assumptions $Da \sim10^{-5}-10^{-4}\ll1$. The small Damköhler number (Da ≪ 1) indicates that under the assumed enzyme loading and representative kinetic parameters, immobilized trypsin does not behave as a perfectly absorbing boundary. Instead, the wall reaction is reaction-limited relative to diffusive supply. In this regime, the apparent digestion efficiency is governed primarily by the effective catalytic activity of immobilized trypsin (including orientation, accessibility, and intrinsic kinetics) rather than by diffusion of Aβ to the enzyme-functionalized channel walls. This Damköhler analysis serves as a scaling check on wall reactivity and does not replace the plug-flow digestion model, which introduces the dimensionless reaction-opportunity parameter (μ). The parameter μ captures the cumulative probability of enzyme–substrate encounters during electrokinetic transit and is determined by reactor geometry (l, d), analyte diffusivity (D_A_), and flow velocity (v). Thus, although the wall reaction itself is not diffusion-limited, the overall digestion efficiency is transport-controlled through residence-time-dependent encounter statistics.

This framework supports the conclusion that deviations from complete digestion observed experimentally are more plausibly attributed to residence-time dispersion and multi-occupancy effects rather than to insufficient diffusive delivery of substrate to the immobilized enzyme layer. Multi-occupancy effects, quantified by the Poisson occupancy parameter (λ), further reduce apparent digestion completeness at higher concentrations by introducing crowding-induced competition during coincident transits.

**9 Estimation of Electric Double Layer Thickness**

The thickness of the electric double layer (EDL) in the nanofluidic reactor was estimated using the Debye length, which characterizes the electrostatic screening distance in an electrolyte solution. The Debye length is given by;

$\lambda{}_{D} =\sqrt{\left( \frac{\varepsilon_{r}\varepsilon_{0}RT}{2F^{2}I} \right)}$	(S11)

### where 𝜀_r_ is the dielectric constant of water, 𝜀_0_ is the permittivity of free space, R is the universal gas constant, T is the absolute temperature, F is the Faraday constant, and I is the ionic strength of the electrolyte. The ionic strength is defined as;

### $\boldsymbol{I=}\left( \frac{\boldsymbol{1}}{\boldsymbol{2}} \right)\boldsymbol{\sum}\boldsymbol{c}_{\boldsymbol{i}}\boldsymbol{z}_{\boldsymbol{i}}^{\boldsymbol{2}}$ (S12)

where, c_i_ is the molar concentration of ion i and z_i_ is the ionic charge. For aqueous solutions at 298 K, the Debye length can be approximated as,

$\lambda{}_{D}\left( nm \right) \approx\frac{0.304}{\surd\left( I\left( M \right) \right)}$ (S13)

The buffer used in this study consisted of 1× PBS supplemented with 1 M KCl. Using an ionic strength of ~1.1 M gives $\lambda{}_{D} \approx0.29 nm$. Thus, the total thickness of opposing double layers is approximately $2\lambda{}_{D} \approx0.58 nm$.

The cross-sectional dimensions of the eINER nanochannel are 250 nm × 250 nm and thus, the combined EDL thickness represents less than 0.3% of the channel width. Therefore, overlap of the electric double layers from opposing channel walls is negligible. Under these high ionic strength conditions, the electroosmotic velocity profile is therefore expected to be nearly uniform across the channel cross section. Consequently, electroosmotic transport within the eINER can be reasonably approximated as plug-like flow, which justifies the use of plug-flow assumptions in the reaction-transport analysis.

**10 Plug-Flow Surface Reaction Model**

Under plug-flow conditions with wall-bound enzymes, the longitudinal concentration of intact Aβ, denoted [A](z), satisfies:

$v \frac{d\left[ A \right]}{dz}=-k_{m} a_{s} F_{A} \left[ A \right]$	(S14)

where [A] is the intact analyte concentration; z is the axial coordinate; k_m_ is the mass-transfer coefficient (m s^−1^); a_s_ is the surface-area-to-volume ratio (m^−1^); and F_A_ is the effective enzyme fraction of the wall (dimensionless). For laminar internal flow;

$k_{m}=\frac{\text{Sh} D_{A}}{d}$ (S15)

where S_h_ is the Sherwood number (dimensionless mass-transfer coefficient) [8]. For fully developed laminar flow in a square channel, Sh ≈ 3.61 under the assumed constant wall-flux boundary condition [9]. For a square channel;

$a_{s}=\frac{4}{d}$	(S16)

Substituting and integrating from z = 0 to l yields:

$\left[ A \right]_{o}=\left[ A \right]_{i}\exp\left( -K \frac{D_{A}F_{A} l}{v d^{2}} \right)$	(S17)

where [A]_i_ is the inlet concentration; [A]_o_ is the outlet concentration; and K = 4Sh = 14.43.

#### 10.1 Dimensionless Digestion Opportunity Parameter

We define;

$\mu=14.43 \frac{D_{A}F_{A} l}{vd^{2}}$	(S18)

where μ is the dimensionless reaction opportunity parameter Using experimental values $\mu\approx129.91 F_{A}$. Thus;

$\frac{\left[ A \right]_{o}}{\left[ A \right]_{i}}=e^{-\mu}$ (S19)

#### 11 Probability of Complete Digestion

Aβ complete digestion requires ≥3 cleavage events. To estimate the probability of complete digestion during a single eINER transit, cleavage events were modeled as a Poisson counting process with mean μ. The probability of achieving ≥3 cleavage events is;

$P\left( \text{complete} \right)=1-e^{-\mu}\left( 1+\mu+\frac{\mu^{2}}{2} \right)$	(S20)

Using $\mu\approx129.91 F_{A}$ gives the values shown in **Table S3**.

**Table S3.** Dependence of digestion completeness on effective enzyme fraction of the wall. Calculated values of the dimensionless digestion-opportunity parameter (μ) and the predicted probability of complete digestion (P_complete_) as a function of the effective enzyme fraction of the wall (F_A_). Increasing F_A_ results in a proportional increase in μ leading to near-unity digestion probability under the modeled transport and reaction conditions.

| **F_A_** | **Μ** | **P_complete_** |
| --- | --- | --- |
| 0.01 | 1.299 | 0.143 |
| 0.10 | 12.991 | 0.9998 |
| 0.50 | 64.954 | ≈1 |
| 1.00 | 129.908 | ≈1 |

With our experimentally relevant transport parameters, the model predicts that complete digestion is readily achieved when the effective enzyme fraction of the wall satisfies $F_{A}\geq0.1 \Rightarrow P_{\text{complete}}\approx0.9998$ and $F_{A}\geq0.5 \Rightarrow P_{\text{complete}}\approx1$. Thus, within realistic F_A_ ranges for a well-functionalized, enzyme-coated nanoreactor wall, the eINER architecture is predicted to support near-quantitative conversion of Aβ to its four tryptic peptide fragments during a single transit (**Figure S4**). However, as seen in **Figure S5**, the probability of complete digestion drops precipitously as the eINER channels get larger.

**Figure S4**. Probability of full digestion as a function of the dimensionless reaction opportunity parameter (μ). The curve represents the modeled probability that a substrate molecule undergoes complete digestion (≥3 cleavage events) within the eINER. As μ increases, the probability rapidly approaches unity, indicating a high likelihood of full digestion under sufficient reaction opportunity. The dashed vertical line marks the experimentally relevant μ value used in this study, showing that the operating conditions fall within a regime where the model predicts near-complete digestion of the analyte molecules.

**Figure S5**. Predicted probability of complete digestion (≥3 cleavages) as a function of the average axial velocity v for nanochannels with channel dimensions from 50 to 2000 nm. The probability was calculated using the Poisson expression P_complete_ = 1 − e^−μ^ (1 + μ + μ^2^/2), where μ = 14.43(D_A_F_A_l)/(vd^2^) represents the dimensionless digestion opportunity parameter. The model shows that digestion probability decreases with increasing velocity, while smaller channel dimensions substantially enhance digestion because μ scales with 1/d^2^. The dashed vertical line indicates the experimentally estimated average axial velocity within the eINER.

**12 Dependence of Analyte Conversion on Longitudinal Velocity and Channel Dimension**

**Figure S6** shows the predicted conversion efficiency as a function of average longitudinal velocity for several representative channel dimensions. The calculations demonstrate that conversion efficiency decreases with increasing velocity because shorter transport times reduce the opportunity for enzymatic cleavage during passage through the reactor. At a fixed velocity, smaller channel dimensions significantly enhance conversion efficiency due to the inverse d^2^ dependence of the reaction-transport parameter. The dashed vertical line in **Figure S6** indicates the experimentally estimated average longitudinal velocity within the eINER.

The model reveals a strong sensitivity of conversion efficiency to channel dimensions, reflecting the 1/d^2^ scaling in the exponential term of the governing expression (see **Eq. S17**). As the channel dimension decreases, analyte molecules experience a greater probability of interacting with the enzyme-functionalized wall resulting in substantially higher conversion efficiencies (**Figure S6**). This behavior highlights the intrinsic advantage of nanoscale confinement in enhancing catalytic interaction between flowing analytes and immobilized enzymes.

**Figure S6**. Predicted conversion efficiency (X), where X = 1 − [A]_o_/[A]_i_ as a function of average axial velocity v for several representative eINER channel dimensions. Calculations were based on the plug-flow surface reaction model. Increasing velocity reduces conversion efficiency because shorter transport times limit enzymatic cleavage during passage through the reactor. Smaller channel dimensions maintain high conversion efficiency due to the inverse d^2^ dependence of the reaction-transport parameter. The dashed vertical line indicates the experimentally estimated average axial velocity in the eINER. An effective enzyme fraction of the wall, F_A,_ of 0.10 was used as a representative value for immobilized enzyme systems accounting for incomplete enzyme accessibility and orientation effects on the surface.

### 13 Feature Importance Analysis and Model Validation of the Random Forest Model

Model performance was further evaluated using stratified five-fold cross-validation. The cross-validation accuracy, balanced accuracy, macro-F1, and weighted-F1 were 99.6 ± 0.9%, 99.7 ± 0.6%, 99.7 ± 0.7%, and 99.6 ± 0.9%, respectively. The close agreement between macro-F1 and weighted-F1 as well as between balanced accuracy and overall accuracy indicates that the unequal numbers of peak pairs among peptide classes did not materially influence the evaluation of classifier performance.

To evaluate the Random Forest model, the permutation feature importance test was computed on the held-out test set using 50 repeats [10]. **Figure S7a** shows that the ToF was the dominant feature contributing to held-out predictive performance. This is followed by normalized peak amplitudes from pores 2 and pore 1. On the other hand, the contributions of dwell times from both pores are minimal relative to the other features. Although dwell times contribute less than ToF and normalized peak amplitudes, they likely provide complementary information. These results highlight an important advantage of dual in-plane nanopores, which enable a discriminative transport-derived feature ToF.

**Figure S7**. Random Forest feature importance and model validation analysis. (**a**) Permutation feature importances estimated on the held-out test set (n_repeats = 50). (**b**) Gini feature importances derived from the trained Random Forest model using the training data.

During training, Gini importance [11] indicates that the Random Forest model relied primarily on the normalized peak amplitudes from pores 1 and 2, followed by ToF and dwell times for both pores (**Figure S7b**). Compared to permutation importance calculated on the held-out test set, the relative contribution of dwell times is higher in the training-derived metric. To further evaluate potential overfitting or data leakage, label-shuffle validation was performed using 200 permutations. The mean cross-validation accuracy was 23.7% ± 1.9%, approaching random classification, compared with a five-fold cross-validation accuracy of 99.6% ± 0.9% obtained using the original class labels. The substantial reduction in accuracy confirms that predictive performance arises from learned structure rather than memorization.

### 14 Single-Molecule Occupancy and Multi-Occupancy Probabilities in the eINER

Molecular loading into the eINER was modeled using Poisson statistics, appropriate for stochastic occupancy in femtoliter-scale volumes [12]. The mean number of molecules in the reactor is;

$\lambda= N_{A}V_{eINER}C$	(S21)

where λ is the mean molecular occupancy (expected number of molecules in the reactor); N_A_ is Avogadro’s number 6.022 × 10^23^ mol^-1^; V_eINER_ is the total eINER volume 1.0553 × 10^-15^ L; and C is the bulk analyte concentration (mol L^-1^). The Poisson probability of observing exactly *n* molecules in the eINER is;

$P\left( n \right)=\frac{\lambda^{n}e^{-\lambda}}{n!}$	(S22)

where P(n) is the probability of observing *n* molecules; *n* is the number of molecules; and e is the base of the natural logarithm. The relevant occupancy probabilities are: (i) Probability that at least one molecule is present: $P\left( \geq1 \right)= 1 - e^{-\lambda}$; (ii) Probability of single-molecule occupancy: $P_{1} = \lambda e^{-\lambda}$; (iii) Probability of multi-occupancy, i.e., two or more molecules: $P(\geq2) = 1 - e^{-\lambda\left( 1+\lambda\right)}$. **Table S4** shows the calculated P values.

**Table S4.** Poisson-based analysis of single-molecule and multi-molecule occupancy within the eINER. At 1 nM (λ = 0.636), 47.0% of reactor volumes are occupied, dominated by single-molecule events (33.7%) with 13.4% multi-occupancy. At 100 pM (λ = 0.0636), overall occupancy decreases to 6.16%, primarily reflecting single-molecule loading (5.96%) with negligible multi-occupancy (0.19%).

| **Aβ Concentration** | **Mean Occupancy (λ)** | **P (≥1) Occupied** | **P₁ Single** | **P (≥2) Multi** |
| --- | --- | --- | --- | --- |
| 1 nM | 0.636 | 47.0% | 33.7% | 13.4% |
| 100 pM | 0.0636 | 6.16% | 5.96% | 0.19% |

### 15 Probability of intact molecules under multi-occupancy

If *n* molecules occupy the reactor and each molecule has an independent probability *p* of remaining undigested, the probability that at least one molecule exits intact is;

$P\left( \geq1 \text{undigested} \mid n \right)=1-\left( 1-p \right)^{n}$	(S23)

Thus, as the weight of higher-order occupancy states (n≥2) increases, the likelihood that at least one molecule “slips through” intact increases even when enzyme density is high. The observed fraction of events assigned by the RF classifier to the intact Aβ class can be modeled as:

$f_{\text{undig}}=f_{\text{single}}+\alpha P\left( \geq2 \right)$ (S24)

where f_undig_ is the fraction of events assigned by the RF classifier to the intact Aβ class; f_single_ is the intrinsic undigested fraction under single-occupancy conditions; P (≥2) is the multi-occupancy probability obtained from Poisson statistics; and α is the crowding-dependent digestion penalty coefficient. Using the RF-assigned intact Aβ event fractions gives $f_{\text{undig}}\left( 1 \text{nM} \right)=0.095$ and $f_{\text{undig}}\left( 100 \text{pM} \right)=0.0033$. Substitution yields $0.095=f_{\text{single}}+\alpha\left( 0.1337 \right)$ and $0.0033=f_{\text{single}}+\alpha\left( 0.00194 \right)$. Solving gives $\alpha\approx0.70$ and $f_{single} \approx0.00195$. At 100 pM, multi-occupancy is negligible (0.19%), and digestion proceeds predominantly under single-molecule conditions yielding a minimal predicted intact Aβ event fraction (~0.20%). At 1 nM, the multi-occupancy probability increases to 13.4%, and crowding-driven competition for immobilized trypsin active sites is consistent with an increased fraction of events assigned by the RF classifier to the intact Aβ class. This analysis demonstrates that incomplete digestion is dominated by occupancy-induced crowding rather than intrinsic enzymatic inefficiency.

### 16 Estimation of −COOH Site Density and Trypsin Loading in the eINER

The −COOH surface density generated by UV/O₃ activation was estimated from published STORM measurements on UV/O₃-activated COC surfaces [13]. Reported site densities were N_5_ ≈ 152 µm^−2^ (5 min) and N_10_ ≈ 203 µm^−2^. For the 6 min UV/O₃ exposure used here, linear interpolation gives;

$\rho_{COOH}= N_{5}+ \frac{(6-5)}{(10-5)}\left( N_{10}-N_{5} \right)= 152+\frac{1}{5}\left( 203-152 \right)=162.2 \text{COOH}\text{ }\mu\text{m}^{\text{-2}}$ (S25)

The eINER consists of square nanochannels with width and depth d = 0.25 µm. For a channel segment of length L, the total internal surface area is $S = \left( 4d \right)L = \left( 4\times0.25 \right)L = L (\mu\text{m}^{\text{2}})$. The total eINER length (two enzyme-input channels plus the full curved channel) was: $L_{\text{total}}=7.5+7.5+1.88496=16.88496 \mu\text{m}$. Thus, the total internal surface area of the eINER is $S_{\text{eINER}}=16.88496 \mu\text{m}^{\text{2}}$. The total number of surfaces −COOH groups in the eINER is $N_{\text{COOH}}=\rho_{COOH}S_{\text{eINER}}=162.2\times16.88496\approx2.74\times10^{3}$.

Trypsin was immobilized via EDC/NHS coupling between surface −COOH groups and enzyme primary amines. Based on a previously published EDC/NHS coupling efficiency range of 10–30%, an effective coupling efficiency of ε = 0.10 (10%), representing the conservative lower end of this range, was used for the estimation [14]. An approximate 1:1 correspondence between activated −COOH sites and immobilized trypsin molecules was assumed [14]; $N_{\text{Trypsin}}={\varepsilon N}_{\text{COOH}}=0.10\times2.74\times10^{3}\approx2.74\times10^{2}$. Therefore, the eINER is estimated to contain ∼2.74 × 10^3^ −COOH sites and ∼2.74 × 10^2^ immobilized trypsin molecules under the stated assumptions.

### 17 Single-Molecule Occupancy in the Flight-Tube Following Digestion

Under the near-quantitative digestion conditions established for the eINER, the fragment concentration entering the sensing channel is approximated as $\left[ C \right]_{\text{frag}}\approx4 \left[ C \right]_{\text{A}\beta}$, where C_frag_ is the total peptide fragment concentration (mol L^−1^) after complete digestion, and C_Aβ_ is the initial bulk concentration of intact Aβ prior to digestion. To evaluate whether detected peak pairs arise predominantly from single molecules, a Poisson occupancy model was applied to the sensing (flight-tube) volume (V_s_). The sensing channel has a cross-section of 50 nm × 50 nm, and the pore-to-pore flight tube length is L_ft_ = 10 μm, giving $V_{s}= 2.5\times{10}^{-17} \text{L} \left( 25 \text{aL} \right)$. The mean occupancy is then given by $\lambda_{s}=N_{A} V_{s} \left[ C \right]_{\text{frag}}$ and the multi-occupancy probability (two or more fragments simultaneously present) is $P\left( \geq2 \right)=1-e^{-\lambda_{s}}\left( 1+\lambda_{s} \right)$. **Table S5** gives the occupancy probability values for the flight tube at two different Aβ concentrations.

**Table S5.** Predicted fragment occupancy in the flight-tube sensing volume after complete digestion of Aβ. Assuming four fragments per Aβ molecule ([C]_frag_ ≈ 4[Aβ]), the mean occupancy (λₛ) and multi-occupancy probability P (≥2) were calculated using Poisson statistics. At 1 nM Aβ, λₛ = 0.060 with 0.174% multi-occupancy, while at 100 pM Aβ, λₛ = 0.0060 with 1.81 × 10^−5^ multi-occupancy, indicating predominantly single-fragment detection conditions.

| **Aβ concentration [Aβ]** | **Fragment concentration [C]_frag_** | **Mean occupancy (λ_s_)** | **Multi-occupancy probability, P (≥2)** |
| --- | --- | --- | --- |
| 1 nM | 4 nM | 0.060 | 0.00174 (0.174%) |
| 100 pM | 400 pM | 0.0060 | 1.81 × 10⁻⁵ |

**References**

1.	Chandrasoma, I. A.; Akabirov, K.; Fateru, O.; Childers, K.; Vaidyanathan, S.; Barros, S. M.; Chibuike, M.; Shivanka, S.; Verber, M.; McKinney, C.; Athapattu, U.; Premarathne, P.; Shiri, F.; Witek, M. A.; Choi, J.; Hall, A. R.; Park, S.; Soper, S. A.,"Plastic Nanofluidic Sensor with a Solid-Phase Bioreactor and Dual Nanopore Reader: Studying Biological Reactions at the Single-Molecule Level," *ACS Applied Materials & Interfaces* 18 (2026): 38600–38614.

2.	Shiri, F.; Choi, J.; Vietz, C.; Rathnayaka, C.; Manoharan, A.; Shivanka, S.; Li, G.; Yu, C.; Murphy, M. C.; Soper, S. A.; Park, S.,"Nano-injection molding with resin mold inserts for prototyping of nanofluidic devices for single molecular detection," *Lab on a Chip* 23 (2023): 4876–4887.

3.	Hedstrom, L.,"Serine Protease Mechanism and Specificity," *Chemical Reviews* 102 (2002): 4501–4524.

4.	Goettig, P.; Brandstetter, H.; Magdolen, V.,"Surface loops of trypsin-like serine proteases as determinants of function," *Biochimie* 166 (2019): 52–76.

5.	Antalis, T. M.; Bugge, T. H.; Wu, Q.,"Membrane-anchored serine proteases in health and disease," *Progress in Molecular Biology & Translational Science* 99 (2011): 1–50.

6.	Thomsen, N. K.; Christensen, U.; Madsen, K.; Sottrup-Jensen, L.,"Interaction between bovine trypsin and a synthetic peptide containing 28 residues of the bait region of human α2-macroglobulin," *European Journal of Biochemistry* 209 (1992): 409–414.

7.	Kawabata, S.-I.; Miura, T.; Morita, T.; Kato, H.; Fujikawa, K.; Iwanaga, S.; Takada, K.; Kimura, T.; Sakakibara, S.,"Highly sensitive peptide-4-methylcoumaryl-7-amide substrates for blood-clotting proteases and trypsin," *European Journal of Biochemistry* 172 (1988): 17–25.

8.	Montagnaro, F. Sherwood (Sh) Number in Chemical Engineering Applications—A Brief Review *Energies* [Online], **2024**, p. 4342.

9.	Bennett, T. D.,"Correlations for Convection in Hydrodynamically Developing Laminar Duct Flow," *Journal of Heat Transfer* 141 (2019).

10.	Föge, N.; Pauly, M.,"Confidence intervals for Random Forest permutation importance with missing data," *Computational Statistics* 41 (2026): p45.

11.	Nembrini, S.; König, I. R.; Wright, M. N.,"The revival of the Gini importance?," *Bioinformatics* 34 (2018): 3711–3718.

12.	Rissin, D. M.; Walt, D. R.,"Digital concentration readout of single enzyme molecules using femtoliter arrays and Poisson statistics," *Nano letters* 6 (2006): 520–523.

13.	ONeil, C. E.; Jackson, J. M.; Shim, S.-H.; Soper, S. A.,"Interrogating Surface Functional Group Heterogeneity of Activated Thermoplastics Using Super-Resolution Fluorescence Microscopy," *Analytical Chemistry* 88 (2016): 3686–3696.

14.	Fischer, M. J. E., Amine Coupling Through EDC/NHS: A Practical Approach. In *Surface Plasmon Resonance: Methods and Protocols*, Mol, N. J.; Fischer, M. J. E., Eds. Humana Press: Totowa, NJ, 2010; pp 55–73.
